# Exercise Versus Standard Therapy in Steatotic Liver Disease Spectrum: A Systematic Review, Meta-Analysis, and Meta-Regression

**DOI:** 10.3390/jcm15145737

**Published:** 2026-07-22

**Authors:** Muhammad Ahsan Asif, Saad Masood, Syed Muhammad Ali Akbar, Muhammad Hamza Khalid, Abdul Rehman, Mahnoor Imran, Arooba Suhaib, Muhammad Ans Asif, Azeem Khalid, Muhammad Sohaib, Arkadeep Dhali, Dushyant Singh Dahiya, Hassam Ali

**Affiliations:** 1 Department of Medicine, Allama Iqbal Medical College, Lahore 54700, Pakistan; 2 Liver Center, Department of Medicine, Beth Israel Deaconess Medical Center, Harvard Medical School, Boston, MA 02215, USA; 3 Department of Medicine, University College of Medicine and Dentistry, Lahore 54000, Pakistan; 4 Department of Surgery, Ghurki Trust Teaching Hospital, Lahore 54000, Pakistan; 5 Internal Medicine, Aiken Regional Medical Centers, Aiken, SC 29801, USA; 6 Department of Internal Medicine, UCHealth Parkview Medical Center, Pueblo, CO 81003, USA; 7 Department of Gastroenterology, Queen’s University Belfast, Belfast BT7 1NN, UK; 8 Division of Gastroenterology, Hepatology & Motility, University of Kansas School of Medicine, Kansas City, KS 66160, USA; 9 Department of Gastroenterology, East Carolina University/Brody School of Medicine, Greenville, NC 27834, USA

**Keywords:** metabolic dysfunction-associated steatotic liver disease, exercise, meta-analysis, liver enzymes, fibrosis indices

## Abstract

**Background:** Metabolic dysfunction-associated steatotic liver disease (MASLD) affects nearly one-fourth of the world population and is closely linked to obesity, metabolic syndrome, and poor diet. We conducted a systematic review and meta-analysis to evaluate the impact of exercise on key clinical outcomes in MASLD patients. **Methods:** A systematic literature search was conducted until 202 September 5, to identify randomized controlled trials (RCTs), post-hoc analyses, and quasi-experimental designs comparing exercise versus standard therapy in MASLD. A random-effects model pooled data as mean differences (MD) or risk ratios (RR) with 95% confidence intervals (CI). Analyses were conducted using R (version 4.5.1). **Results:** Our meta-analysis included 21 RCTs, 4 post-hoc analyses, and 1 quasi-experimental study (1124 patients; mean age 52.24 ± 7.24 years). Exercise reduced BMI (MD −0.82), serum cholesterol (MD −14.31), ALT (MD −6.56), AST (MD −5.32), FIB-4 (MD −0.19), and NAFLD fibrosis score (MD −0.59), and increased HDL (MD 4.36) versus standard therapy. Weight (MD −2.11) and HOMA-IR (MD −0.59) were not significantly different. Meta-regression showed age increased HOMA-IR (estimate 0.032) and higher BMI reduced HDL (estimate −0.71). **Conclusions:** Exercise significantly improves key clinical outcomes in MASLD, including BMI, cholesterol, liver enzymes, and fibrosis markers, supporting physical activity as a core component of MASLD management.

## 1. Introduction

Metabolic dysfunction-associated steatotic liver disease (MASLD), formerly termed nonalcoholic fatty liver disease (NAFLD), is the hepatic component of metabolic syndrome, and is defined by the accumulation of more than 5% liver fat in individuals without significant alcohol consumption, viral hepatitis, or other specific liver disorders [1,2]. It has a prevalence of nearly 25% and is one of the most common causes of chronic liver disease globally, creating a major healthcare challenge [3]. Although heavily studied in the United States, MASLD affects diverse populations worldwide, with reported prevalence ranging between 15% and 35%, highlighting its broad international impact [4,5]. While ongoing research seeks to clarify the genetic factors driving abnormal hepatic fat deposition, increasing evidence suggests that physical inactivity and the widespread adoption of Western dietary patterns have significantly contributed to its growing incidence, particularly in low- and middle-income regions [6,7]. In 2023, NAFLD was reclassified as MASLD to better reflect its metabolic roots and reduce stigmatization, marking an important shift in both clinical practice and research [7]. Similarly, metabolic dysfunction-associated steatohepatitis (MASH), the progressive inflammatory form of MASLD, replaced the term nonalcoholic steatohepatitis (NASH).

MASLD has rapidly emerged as the second leading cause of end-stage liver disease and is associated with a growing number of hepatocellular carcinoma (HCC) cases. As a multisystem metabolic disorder, MASLD is closely linked to obesity, insulin resistance, and dyslipidemia, core components of metabolic syndrome, and leads to liver-related morbidity and mortality. The clinical and economic burden of MASLD is projected to rise substantially in the coming years, underscoring the importance of increasing awareness and implementing earlier and more effective interventions [7,8].

A sedentary lifestyle with excessive caloric intake leads to the development and progression of MASLD, largely through promoting weight gain and worsening insulin resistance. Increasing physical activity and reducing sedentary behavior have been identified as key therapeutic strategies. In the absence of widely effective pharmacologic treatments, lifestyle modification, particularly exercise, is central to disease management [9,10]. Current guidelines from the American Association for the Study of Liver Diseases (AASLD) advocate for weight reduction through a hypocaloric diet or increased physical activity [11,12]. However, evidence specifically isolating the effects of exercise on liver fat independent of dietary change remains limited, highlighting the need for further investigation in this area.

Our study incorporated randomized control trials, post-hoc analyses, and quasi-experimental studies to find the effects of exercise on metabolic and hepatic outcomes in MASLD. In addition, to make the findings robust, we employed sensitivity analyses and meta-regression.

## 2. Methods

### 2.1. Literature Review and Search Strategy

This systematic review and meta-analysis was conducted according to the Preferred Reporting Items for Systematic Reviews and Meta-Analyses (PRISMA) guidelines and the Cochrane Handbook for Systematic Reviews of Interventions [13,14]. The PRISMA checklist can be found in Table S1. The study protocol can be found on the International Prospective Register of Systematic Reviews (PROSPERO) with the ID number CRD420251238047.

We searched PubMed, Cochrane Central Register of Controlled Trials (CENTRAL), Scopus, and Clinicaltrials.gov for suitable articles from inception till 29 September 2025 using the search items “metabolic dysfunction-associated steatotic liver disease” and “non-alcoholic fatty liver disease”. The complete search strategy can be found in the supplemental file. Boolean and proximity operators were used to amalgamate the keywords and indexing terms that had truncation.

### 2.2. Eligibility Criteria

The inclusion criteria for this study consisted of (1) patients with MASLD/MASH who are older than 18 years old. (2) Randomized controlled trials (RCTs) and propensity-matched observational studies. (3) Articles published in the English language.

The exclusion criteria consisted of studies other than RCTs and propensity-matched observational studies, such as case reports, case series, commentaries, review articles, letters to the editors, meta-analyses and non-peer reviewed articles. Furthermore, we excluded studies that did not meet the inclusion criteria or did not have a comparison group.

### 2.3. Outcomes

The primary outcome of the study includes changes from baseline in basal metabolic index (BMI), weight, serum cholesterol, ALT levels, AST levels, fibrosis-4 index (FIB-4), NAFLD fibrosis score (NFS).

The secondary outcomes includes changes from baseline in visceral adipose fat, peak VO_2_ levels, fasting blood glucose levels, HbA1c, homeostatic model assessment of insulin resistance (HOMA-IR), triglyceride levels, LDL level, HDL level, and liver associated parameters including intrahepatic triglyceride, magnetic resonance imaging proton density fat fraction (MRI-PDFF) liver fat, ≥30% relative reduction in MRI-PDFF.

### 2.4. Study Screening and Data Extraction

The initial screening and assessment of the articles based on titles and abstracts were done by two independent reviewers. Following this, the same two reviewers evaluated the full-text articles to determine the eligibility based on the inclusion criteria. In case of a conflict, it was resolved either by mutual consensus or a third reviewer. Data was extracted on Google Sheets by five independent reviewers and was then cross-checked by four independent reviewers to resolve any issues. Data extracted from each study included study characteristics, participant baseline characteristics, and outcome data.

### 2.5. Statistical Analysis

All outcomes were pooled with 95% confidence intervals (CIs) using a random-effects model. Risk ratios (RRs) with 95% CIs were calculated for dichotomous data, and mean differences (MDs) were used for continuous data. Statistical significance was set at *p* < 0.05, and all analyses were performed in R (version 4.5.1) using packages “meta”, “dplyr”, and “metasens”. Heterogeneity was measured using the I^2^ statistic, with values above 50% indicating substantial heterogeneity and values over 75% indicating considerable heterogeneity. Results were visually represented with forest plots. Publication bias was assessed using Egger’s regression test and Luis Furuya-Kanamori (LFK) Index, and visualized using funnel plots and Doi plots. A leave-one-out sensitivity analysis was conducted to evaluate the impact of individual studies on the results. A univariable meta-regression (mixed effects model) was undertaken to further explore the heterogeneity using baseline age and BMI as covariates.

### 2.6. Quality Assessment

The quality of the included studies was evaluated using the Cochrane Risk of Bias tools. For randomized controlled trials, the revised Cochrane Risk of Bias tool (RoB-2) was applied [15], in which bias is evaluated as a judgment (low, some concerns, or high) across five domains: the randomization process, deviations from intended interventions, missing outcome data, measurement of the outcome, and selection of the reported result. For cohort and other observational studies, the Risk of Bias in Non-randomized Studies of Interventions (ROBINS-I V2) [16] tool was used, which assesses bias across seven domains: confounding, selection of participants, classification of interventions, deviations from intended interventions, missing data, measurement of outcomes, and selection of reported results. For both tools, the risk of bias for each domain was categorized according to the respective assessment criteria, and an overall judgment was assigned based on domain-level evaluations.

## 3. Results

### 3.1. Search Results and Study Selection

**Figure 1:**
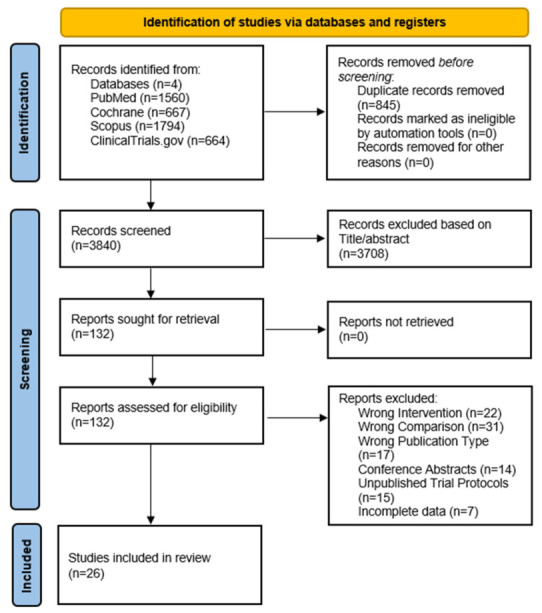
PRISMA flow diagram summarizing the study selection process.

A total of 4685 articles were initially identified through four database searches. After removing 845 duplicates, screening based on title and abstract was done, which left us with 132 articles for full text search review. After completion of full text review, 106 articles were excluded. Finally, 26 studies met the inclusion criteria and were listed in our meta-analysis [17,18,19,20,21,22,23,24,25,26,27,28,29,30,31,32,33,34,35,36,37,38,39,40,41,42]. Major exclusion criteria included severe life-limiting illnesses (such as cancer or renal failure), neuromuscular or orthopedic limitations, hepatitis B/C or other chronic liver diseases, genetic disorders, prior weight-loss treatments, uncontrolled diabetes, excess alcohol consumption, inability to perform regular exercise, and current hormonal therapy. The comprehensive process of literature search and study selection is demonstrated in the PRISMA flow diagram (Figure 1).

### 3.2. Study Characteristics

Our meta-analysis included 21 RCTs, 4 post-hoc analyses, and 1 quasi-experimental study, encompassing a total population of 1124 patients. The pooled mean age of the study population was 52.24 ± 7.24 years. The detailed study and baseline characteristics of the included studies are demonstrated in Table 1 and Table 2.

### 3.3. Risk of Bias

Assessment of the risk of bias is shown in Supplementary Figures S1 and S2. ROB-2 assessment was used for 25 studies; 3 studies had an overall low risk of bias, while 24 studies showed some overall concerns. The majority had concerns in domains 1 and 2 in the bias assessment. The concerns in Domain 1 were mainly related to insufficient reporting of allocation concealment or incomplete description of the randomization procedure, which may introduce potential selection bias. In Domain 2, several studies had limited information regarding adherence to assigned interventions or lack of blinding, raising the possibility of deviations from intended treatments that could influence outcome measurements. Robins-1 was used for the assessment of one study, which revealed an overall moderate risk of bias (Supplementary Figures S1–S4).

### 3.4. Outcomes and Heterogeneity Assessment

#### 3.4.1. Primary Outcomes

##### Mean Change in BMI

**Figure 2:**
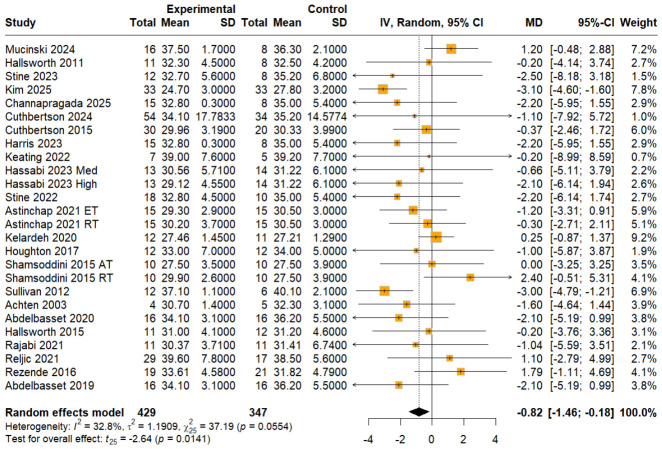
Forest plot showing the pooled mean difference in change in body mass index (BMI) [17,18,19,20,21,22,23,24,25,26,27,29,31,32,33,34,35,36,37,39,40,41,42].

Change in BMI post-intervention was reported by 23 studies. A combined analysis of the data revealed a statistically significant reduction in BMI in the exercise group compared to the placebo group (MD= −0.82; 95% CI: −1.46 to −0.18; I^2^ = 32.8%; *p* = 0.0141), as illustrated in Figure 2.

Leave one out sensitivity analysis showed omitting Kim 2025 [19] reduced the heterogeneity to 10% with *p* value = 0.0562, suggesting Kim 2025 [19] was the primary driver for observed efficacy for BMI (Supplementary Figure S5a).

Additionally, subgroup analysis by exercise modality showed a statistically significant reduction in BMI exclusively in the aerobic exercise group; the significant test for subgroup differences (*p* = 0.0052) suggests variation in effect across exercise modalities (Supplementary Figure S5b).

##### Mean Change in Weight

**Figure 3:**
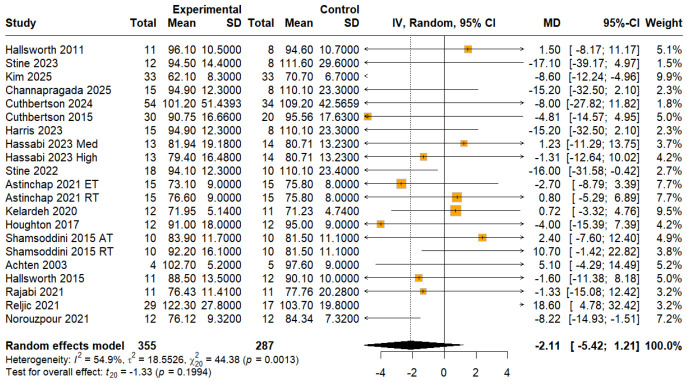
Forest plot showing the pooled mean difference in change in body weight [18,19,20,21,22,23,25,26,27,29,31,32,34,36,37,38,41,42].

Change in weight post-intervention was reported by 18 studies. A pooled analysis revealed a statistically non-significant weight reduction in the intervention group (MD = −2.11; 95% CI: −5.42 to 1.21; I^2^ = 54.9%; *p* = 0.1994), as illustrated in Figure 3.

A sensitivity analysis excluding the non-randomized study (Norouzpur 2021) [38] was conducted; its omission did not materially alter the overall findings (I^2^ = 54.4%; *p* = 0.3294), as shown in Supplementary Figure S6a.

Additionally, subgroup analysis by exercise modality demonstrated a statistically significant reduction in weight in the aerobic exercise group only; the test for subgroup differences was also significant (*p* = 0.0105), indicating a differential effect across exercise modalities (Supplementary Figure S6b).

##### Mean Change in Serum Cholesterol

**Figure 4:**
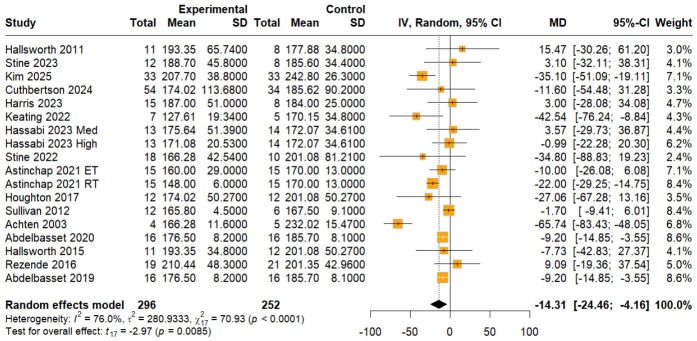
Forest plot showing the pooled mean difference in the change in serum cholesterol [18,19,21,23,24,25,26,27,31,33,34,35,36,39,40,42].

A change in serum cholesterol was reported by 16 studies. A pooled analysis of the studies revealed a statistically significant reduction in serum cholesterol in the experimental group (MD = −14.31; 95% CI: −24.46 to −4.16; I^2^ = 76%; *p* = 0.0085), as illustrated in Figure 4. Leave-one-out sensitivity analysis showed omitting Achten 2003 [34] reduced the heterogeneity to 54.1% with *p* value = 0.0038, suggesting it was a source of variance (Supplementary Figure S7a).

Additionally, a subgroup analysis by exercise modality was performed and demonstrated statistically significant reduction in serum cholesterol in the HIIT and Resistance Training subgroups; the test for subgroup differences was also statistically significant (*p* = 0.0445), indicating variation across different exercise modalities (Supplementary Figure S7b).

##### Mean Change in Serum ALT Levels

**Figure 5:**
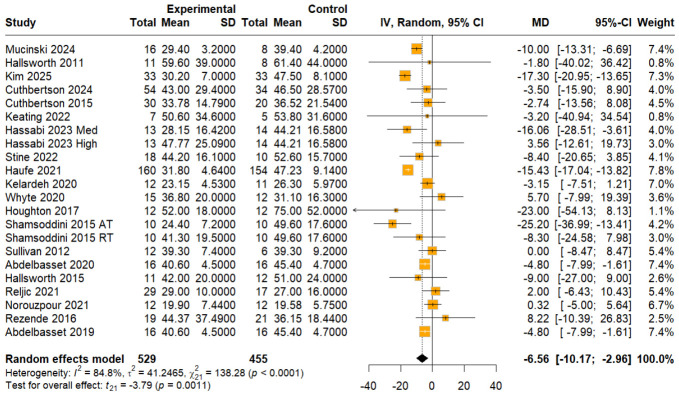
Forest plot showing the pooled mean difference in change in alanine aminotransferase (ALT) levels [17,18,19,21,22,24,25,26,28,29,30,31,32,33,35,36,38,39,40,41].

Mean change in serum ALT levels was reported by 20 studies. A pooled analysis of the studies revealed a statistically significant reduction in serum ALT levels in the experimental group (MD = −6.56; 95% CI: −10.17 to −2.96; I^2^ = 84.8%; *p* = 0.0011), as illustrated in Figure 5.

Leave-one-out sensitivity analysis showed that omitting any single study does not contribute substantially to the significance of the reduction (Supplementary Figure S8a).

Additionally, a sensitivity analysis excluding non-randomized studies (Norouzpur 2021) [38] was performed; the results remained statistically significant and comparable to the overall analysis (I^2^ = 83.6%; *p* = 0.0008), as shown in Supplementary Figure S8b.

A subgroup analysis based on exercise modalities demonstrated a statistically significant reduction in serum ALT levels in both the HIIT and Resistance Training subgroups; however, the test for subgroup differences was not significant (*p* = 0.6578), as demonstrated in Supplementary Figure S8c.

##### Mean Change in Serum AST Levels

**Figure 6:**
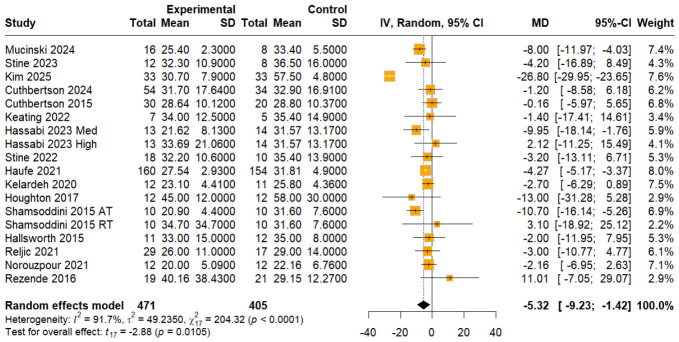
Forest plot showing the pooled mean difference in change in aspartate aminotransferase (AST) [17,19,21,22,24,25,26,28,29,31,32,36,38,39,41,42].

Mean change in serum AST levels was reported by 16 studies. A pooled analysis of the studies revealed a statistically significant reduction in serum AST levels in the experimental group (MD = −5.32; 95% CI: −9.23 to −1.42; I^2^ = 91.7%; *p* = 0.0105), as illustrated in Figure 6. Leave-one-out sensitivity analysis showed omitting Kim 2025 [19] reduced the heterogeneity to 20.3% with *p* value = 0.0002, suggesting it was a source of variance (Supplementary Figure S9a).

Additionally, a sensitivity analysis excluding non-randomized studies (Norouzpur 2021) [38] was performed; the results remained comparable to the overall analysis and were statistically significant (I^2^ = 92.1%; *p* = 0.0125), as shown in Supplementary Figure S9b.

A subgroup analysis by exercise modality demonstrated statistically significant reduction in serum AST levels in the HIIT subgroup only; however, the test for subgroup differences was not significant (*p* = 0.4140), as demonstrated in Supplementary Figure S9c.

##### Mean Change in Fibrosis-4 Index (FIB-4) Score

**Figure 7:**
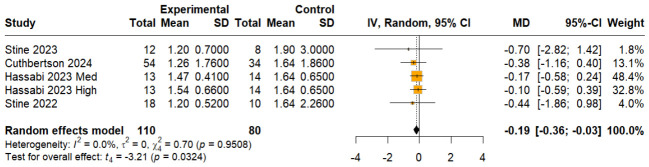
Forest plot showing the pooled mean difference in change in fibrosis-4 (FIB-4) score [21,25,26,42].

Mean change in FIB-4 score, a marker of liver fibrosis, was reported by 4 studies. A pooled analysis of the studies revealed a statistically significant reduction in FIB-4 score in the experimental group (MD = −0.19; 95% CI: −0.36 to −0.03; I^2^ = 0.0%; *p* = 0.0324), as illustrated in Figure 7.

##### Mean Change in NAFLD Fibrosis Score (NFS)

**Figure 8:**
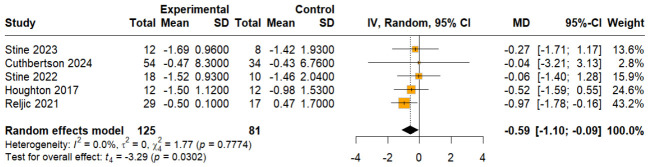
Forest plot showing the pooled mean difference in change in NAFLD fibrosis score (NFS) [21,26,31,41,42].

The mean change in NFS score was reported by 5 studies. A pooled analysis of the studies revealed a statistically significant reduction in NFS score in the experimental group (MD = −0.59; 95% CI: −1.10 to −0.09; I^2^ = 0.0%; *p* = 0.0302), as illustrated in Figure 8.

#### 3.4.2. Secondary Outcomes

##### Mean Change in Visceral Adipose Fat Tissue (VAT)

**Figure 9:**
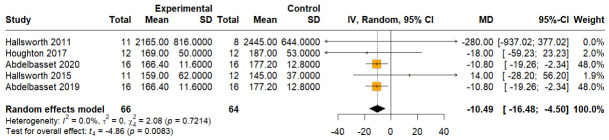
Forest plot showing the pooled mean difference in change in visceral adipose tissue (VAT) [18,31,35,36,40].

Mean change in visceral adipose fat was reported by 5 studies. A pooled analysis of the studies revealed a statistically significant reduction in VAT in the experimental group (MD = −10.49; 95% CI: −16.48 to −4.50; I^2^ = 0.0%; *p* = 0.0083), as illustrated in Figure 9.

##### Mean Change in Peak VO_2_ Levels

**Figure 10:**
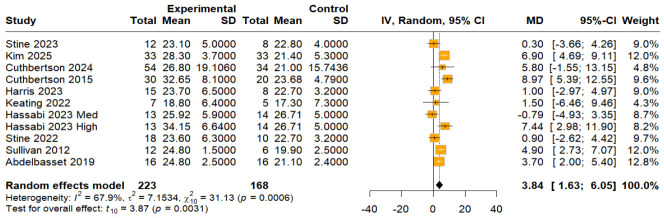
Forest plot showing the pooled mean difference in change in peak oxygen consumption (peak VO_2_) [19,21,22,23,24,25,26,33,40,42].

Mean change in peak VO_2_ levels was reported by 10 studies. A pooled analysis of the studies revealed a statistically significant improvement in peak VO_2_ levels in the experimental group (MD = 3.84; 95% CI: 1.63 to 6.05; I^2^ = 67.9%; *p* = 0.0031), as illustrated in Figure 10.

Leave one out sensitivity analysis showed that omitting any single study does not contribute substantially to the significance of overall benefit. In addition, no single study was identified as the primary source of heterogeneity (Supplementary Figure S10).

##### Mean Change in Fasting Blood Glucose Levels

**Figure 11:**
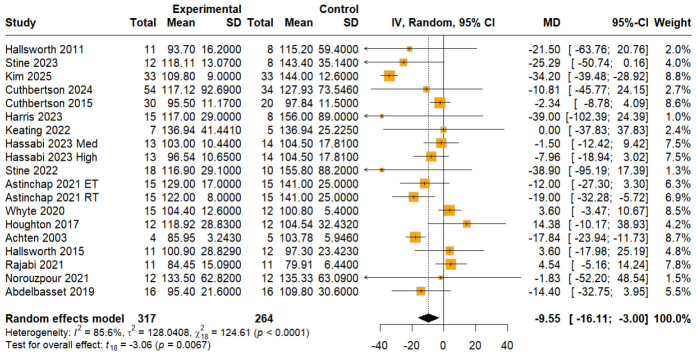
Forest plot showing the pooled mean difference in change in fasting blood glucose levels [18,19,21,22,23,24,25,26,27,30,31,34,36,37,38,40,42].

Mean change in fasting blood glucose levels was reported by 17 studies. A pooled analysis of the studies revealed a statistically significant reduction in fasting blood glucose levels in the experimental group (MD = −9.55; 95% CI: −16.11 to −3.00; I^2^ = 85.6%; *p* = 0.0067), as illustrated in (Figure 11). Leave-one-out sensitivity analysis showed omitting Kim 2025 [19] reduced the heterogeneity to 59.3% with a *p*-value of 0.0188, suggesting that the study was a source of variance (Supplementary Figure S11a).

Additionally, a sensitivity analysis omitting non-randomized studies (Norouzpur 2021) [38] was performed but the results remained statistically significant and comparable to the overall findings (I^2^ = 86.3%; *p* = 0.0081), as shown in Supplementary Figure S11b.

A subgroup analysis based on exercise modality was also performed and revealed statistically significant reduction in fasting blood glucose levels, only in the aerobic exercise group, and the test for subgroup differences was also significant (*p* = 0.0003), as illustrated in Supplementary Figure S11c.

##### Mean Change in HbA1c

**Figure 12:**
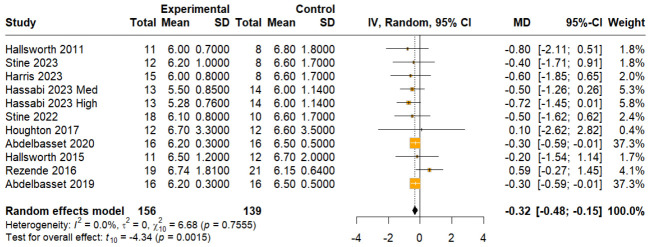
Forest plot showing the pooled mean difference in change in hemoglobin A1c (HbA1c) levels [18,23,25,26,31,35,36,39,40,42].

Mean change in HbA1c levels was reported by 10 studies. A pooled analysis of the studies revealed a statistically significant reduction in HbA1c levels in the experimental group (MD = −0.32; 95% CI: −0.48 to −0.15; I^2^ = 0.00%; *p* = 0.0015), as illustrated in Figure 12.

Additionally, a subgroup analysis was performed based on exercise modality, which demonstrated a statistically significant reduction in HbA1c in the HIIT group, however, the test for subgroup differences was not statistically significant (Supplementary Figure S12).

##### Mean Change in HOMA-IR

**Figure 13:**
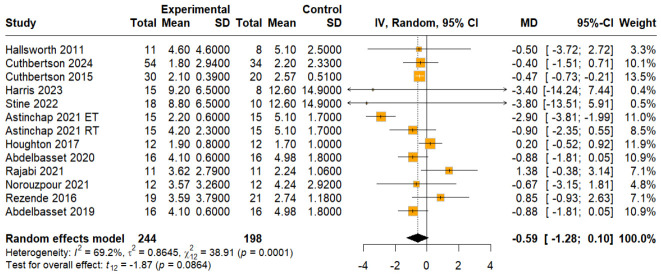
Forest plot showing the pooled mean difference in change in homeostatic model assessment of insulin resistance (HOMA-IR) [18,21,22,23,26,27,31,35,37,38,39,40].

Mean change in HOMA-IR levels was reported by 12 studies. A pooled analysis revealed a statistically non-significant HOMA-IR reduction in the intervention group (MD = −0.59; 95% CI: −1.28 to 0.10; I^2^ = 69.2%; *p* = 0.0864), as illustrated in Figure 13.

Leave-one-out sensitivity analysis revealed that exclusion of Astinchap 2021 (ET) [27] resulted in significant reduction in heterogeneity from 69.2% to 8.4% with the results being statistically significant (*p* = 0.0266), suggesting that the study was a source of variance (Supplementary Figure S13a).

Additionally, a sensitivity analysis excluding non-randomized studies (Norouzpur 2021) [38] was performed, however, the results were comparable to the overall findings (I^2^ = 71.7%; *p* = 0.1140), as shown in Supplementary Figure S13b.

A subgroup analysis on exercise modality was also performed but did not reveal any statistically significant difference among the subgroups (Supplementary Figure S13c).

##### Mean Change in Serum Triglycerides

Mean change in serum triglycerides levels was reported by 19 studies. A pooled analysis revealed a statistically significant reduction in serum triglycerides levels in the intervention group (MD = −22.40; 95% CI: −34.51 to −10.30; I^2^ = 58.5%; *p* = 0.0010), as illustrated in (Supplementary Figure S14). Leave one out sensitivity analysis revealed that eliminating Achten 2003 [27] resulted in significant reduction in heterogeneity to 25.3% with *p* value of 0.0001, suggesting that the study was a source of variance (Supplementary Figure S14a).

Additionally, a sensitivity analysis omitting non-randomized studies (Norouzpur 2021) [38] was performed, however, the results remained statistically significant and comparable to the overall findings (I^2^ = 60.1%; *p* = 0.0030), as shown in Supplementary Figure S14b.

A subgroup analysis based on exercise modality was also performed, and revealed a statistically significant reduction in serum triglycerides in the HIIT and Resistance Exercise subgroups, and the test for subgroup differences was also statistically significant (*p* = 0.0445), as demonstrated in Supplementary Figure S14c.

##### Mean Change in HDL Level

Mean change in HDL levels was reported by 14 studies. A pooled analysis revealed a statistically significant increase in HDL level in the intervention group (MD = 4.36; 95% CI: 2.29 to 6.43; I^2^ = 53.5%; *p* = 0.0004), as illustrated in (Supplementary Figure S15). Leave-one-out sensitivity analysis showed that omitting Kim 2025 [19] reduced the heterogeneity to 0.00% with *p* value = 0.0004, suggesting that the study was a source of variance (Supplementary Figure S15a).

##### Mean Change in LDL Levels

Mean change in LDL levels was reported by 13 studies. A pooled analysis revealed a statistically non-significant LDL level reduction in the intervention group (MD = −9.62; 95% CI: −19.57 to 0.33; I^2^ = 84.8%; *p* = 0.0569), as illustrated in Supplementary Figure S16. Leave-one-out analysis was done to check for heterogeneity and statistical significance. Eliminating Sullivan 2012 [33] resulted in slightly increased heterogeneity to 85.3% with the *p* value of 0.0384 showing statistical significance. A similar trend was observed by eliminating Cuthbertson 2015 [22] with heterogeneity of 85.5% and *p*-value of 0.0444, showing statistical significance, suggesting these studies as possible sources of variance (Supplementary Figure S16a).

##### Mean Change in Liver-Associated Parameters

5 Studies reported mean change in intrahepatic triglyceride levels, 5 studies reported mean change in MRI-PDFF liver fat, and 4 studies reported mean change of ≥30% relative reduction in MRI-PDFF. Pooled analysis of reported studies revealed a statistically non-significant intrahepatic triglyceride level reduction in the intervention group (MD = −3.98; 95% CI: −14.23 to 6.26; I^2^ = 80.8%; *p* = 0.3409), as illustrated in Supplementary Figure S17, a statistically significant reduction in MRI-PDFF liver fat in the intervention group (MD = −5.25; 95% CI: −8.55 to −1.95; I^2^ = 0.00%; *p* = 0.0115), as illustrated in Supplementary Figure S18, and a statistically significant ≥ 30% relative reduction in MRI-PDFF in the intervention group (MD = 2.43; 95% CI: 2.18 to 2.70; I^2^ = 0.00%; *p* = 0.0001), as illustrated in Supplementary Figure S19. Leave-one-out sensitivity analysis showed that omitting Keating 2022 [24] for intrahepatic triglycerides levels reduced the heterogeneity to 0.00% with *p* value = 0.0242, suggesting that the study was a source of variance (Supplementary Figure S17a).

### 3.5. Publication Bias

Publication bias was assessed using Egger’s test and LFK Index, and visualized using funnel plots and Doi plots. Funnels plots showed no substantial publication bias except in serum LDL-C, for which the Egger’s test was also significant (*p* = 0.0490). LFK indices for Doi Plots showed major asymmetry in some outcomes particularly FIB-4 (LFK = −4.88), MRI-PDFF (LFK = −2.98), >30% reduction in MRI-PDFF (LFK = −2.16), NFS (LFK = 2.91), VAT (LFK = −4.65) and intrahepatic triglycerides (LFK = −2.16). These elevated indices suggest that the small study effects may be an influential factor for pooled results for liver fibrosis and fat reduction, needing cautious interpretation.

### 3.6. Meta-Regression

To explore the unresolved heterogeneity, a univariable meta-regression was performed using age and baseline BMI as covariates. Age was not a significant source of heterogeneity for most outcomes, including mean change in weight, cholesterol, HDL levels, LDL levels, VO_2_ peak, AST levels, ALT levels, and fasting blood glucose levels, where the *p*-value was >0.05. However, age accounted for the majority of heterogeneity in HOMA-IR (R^2^ = 62.41%, I^2^ = 54.76%) with *p* value of 0.018. Mean age showed a positive association with HOMA-IR (estimate = 0.032, SE = 0.012, *p* = 0.018, 95% CI: 0.007–0.058), demonstrating that increased age is associated with higher HOMA-IR levels. Age accounted for all the heterogeneity in serum triglycerides (R^2^ = 100%, I^2^ = 0%), however, this did not reach statistical significance (*p* = 0.058).

Similarly, BMI was not a significant source of heterogeneity for most outcomes, including mean change in weight, cholesterol, LDL levels, VO_2_ peak, AST levels, ALT levels, fasting blood glucose levels, and HOMA-IR, where *p* value was >0.05. Baseline BMI accounted for all the heterogeneity in Serum HDL levels (R^2^ = 100%, I^2^ = 0) with a *p*-value of 0.0009. Mean BMI demonstrated a strong negative association with HDL levels (estimate = −0.71, SE = 0.17, t = −4.22, *p* = 0.0009, 95% CI: −1.07 to −0.35), showing that with a rise in BMI per unit, the serum HDL levels tend to decrease by 0.71 units.

## 4. Discussion

This systematic review and meta-analysis consisted of 26 articles (21 randomized controlled trials, four post-hoc studies of randomized trials and one quasi-experimental study), including 1124 adults with MASLD. The data provides one of the most comprehensive evaluations to date of the effect of structured exercise interventions on cardiopulmonary, hepatic, metabolic, and anthropometric variables in adults with metabolic dysfunction-related steatotic liver disease (MASLD). Altogether, the results indicate that exercise enhances various clinically significant outcomes and justifies its use as one of the primary non-pharmacological treatments for MASLD.

BMI, serum cholesterol, ALT, AST, FIB-4 score, and NAFLD fibrosis scores all demonstrated marked reduction, reaching statistical significance. The findings of this meta-analysis distinguish between outcomes that reached statistical significance and those demonstrating clear clinical relevance. Exercise interventions resulted in a statistically significant but modest reduction in BMI (MD = −0.82, *p* < 0.05). While the manuscript does not explicitly define a specific clinical threshold for absolute BMI reduction in MASLD patients, this small change is likely more representative of a shift in body composition, specifically the depletion of adiposity and increase in lean muscle mass than a substantial loss of total body weight. In contrast, the impact on hepatic fat was more profound with a statistically significant reduction in MRI-PDFF liver fat and a significant ≥ 30% relative reduction in MRI-PDFF. This ≥30% relative reduction in MRI-PDFF is of importance as it is recognized in the literature as a clinically significant change and a sensitive indicator of treatment response in MASLD. This aligns with past experiences, which reveal that exercise, without dietary interventions, hardly yields significant weight loss to the body; instead, it selectively depletes adiposity, increases skeletal muscle mass, and enhances metabolic flexibility [43,44]. The lack of statistically significant weight loss (MD = −2.11, *p* = 0.1994) further supports this interpretation, that exercise can decrease the fat mass while increasing lean muscle mass. A standard weighing scale cannot detect it because it changes the body composition. Golabi et al. [45] reported that individuals who combined dietary restrictions with physical exercise had a higher percentage reduction in body fat than those who exercised alone. Sullivan et al. [33] and Hallsworth et al. [18,36] also discovered that exercise decreases body fat percentage even in the absence of weight loss. This pattern is further supported by the notable reduction in the visceral adipose tissue (VAT) in our review as it is a key indicator of hepatic inflammation, insulin resistance, and fibrosis progression [43].

Our analysis showed that while total body weight remained largely unchanged, the VAT decreased significantly. The difference suggests that exercise contributed to preserve or ever increase lean skeletal muscle mass which balanced out the fat lost on the scale. This is important observation because sarcopenic obesity is common in MASLD and it worsens the metabolic health [46,47,48]. Skeletal muscles are the main sites in body where insulin disposes of glucose and also helps in breaking down fats. Therefore the cross talks between the liver and the healthy muscle is central to the exercise [48,49]. This explains how structured exercise can substantially clear out intrahepatic fat and improve liver health even when the patient does not show major weight loss on a standard weighing scale.

Serum ALT and AST levels were also reduced in our study, supporting the idea that exercise helps to reduce hepatocellular injury and inflammation in the liver. A meta-analysis conducted to identify improvements in both ALT and AST after exercise-based interventions reported similar results [50]. Similar results were also observed in studies by Nam et al. and Sagi et al., in which exercise-based interventions improved serum ALT levels [5,8].

Importantly, our results showed a significant improvement in FIB-4 and NAFLD fibrosis scores, advocating the impact of exercise on modulating fibrogenic pathways. However, it is critical to clarify that these findings reflect changes in the non-invasive surrogate indices rather than direct evidence of altered liver architecture. We acknowledge that true fibrosis regression can only be confirmed via direct histological outcome such as liver biopsy, which remains the gold standard for assessment and was not the primary data source for these specific pooled estimates. Consequently, while these improvements are clinically encouraging, they should be interpreted cautiously as markers of reduced fibrotic risk rather than definitive proof of histological regression [7,44].

Specifically regarding these fibrosis outcomes, it is important to note that both the FIB-4 and NAFLD fibrosis scores rely on liver enzymes (AST and ALT). Exercise causes rapid reduction in these enzymes by reducing the acute liver inflammation, the observed improvement in our analysis may partly reflect a drop in hepatic inflammation rather than the true regression of fibrotic scarring. Recent studies have also emphasized this limitation, noting that non-invasive fibrosis scores can be strongly influenced by liver enzyme changes and therefore should be interpreted with caution when evaluating exercise interventions in MASLD [51,52].

While the aforementioned MRI-PDFF data highlighted robust, clinically significant reductions in hepatic fat, intrahepatic triglycerides measured by other modalities did not significantly improve in the overall pooled analysis. Nonetheless it is important to emphasize that MRI-PDFF is a sensitive, quantitative variable that can identify alterations in hepatic fat much more effectively than conventional biochemical measures and ultrasonography. Prior studies also found significant reductions in hepatic fat in patients with MASLD who underwent structured exercise programs and were later evaluated by MRI [53,54].

Exercise is also attributed to meaningful changes in key metabolic markers such as fasting glucose, HbA1c, triglycerides, and HDL cholesterol. These changes are biologically plausible and align with evidence-based mechanistic pathways. Exercise increases GLUT-4 translocation in skeletal muscle, improves mitochondrial oxidative capacity, promotes fatty acid β oxidation, and improves hepatic insulin sensitivity by lowering hepatic lipid intermediates [43,55].

A noteworthy observation in our analysis of metabolic markers is the emergence of a lipoprotein paradox. Despite significant drops in total cholesterol and triglycerides accompanied by an increase in HDL cholesterol, the decline in LDL cholesterol did not reach statistical significance. Even though we did not measure the remnant cholesterol in our analysis this pattern show that exercise improves lipid metabolism mainly by clearing triglyceride rich remnant lipoproteins, rather than directly lowering the LDL particles [56,57]. Remnant cholesterol, which is total cholesterol minus HDL cholesterol and LDL cholesterol, is increasingly recognized as an independent risk factor for both cardiovascular disease and liver toxicity in patients with MASLD [57,58]. Therefore the lipids improvements we found likely reflect an enhanced clearance of these harmful remnant lipoproteins. This highlights a major clinical benefit of exercise for MASLD patients, even if traditional LDL levels do not change drastically.

One outlier study [24] influenced the pooled estimate of intrahepatic triglycerides. Despite this statistical insignificance, sensitivity analysis and meta-regression indicated that the presence of a single influential Study distorted the initial results. Similarly, another study [27] was found to be an outlier, and a statistically significant improvement in HOMA-IR was observed after removing it from the analysis, indicating that it was masking the beneficial effects of exercise on HOMA-IR.

Cardiorespiratory fitness improved significantly, as indicated by an increase in VO_2_. The finding is significant, as VO_2_ is one of the strongest predictors of long-term survival in patients with metabolic and cardiovascular disorders. A 10% decrease in VO_2_ correlates with a 15% increase in all-cause mortality and an increased risk of cardiovascular mortality [41,59,60]. Thus, the substantial increase in VO_2_ observed in the intervention group indicates a decrease in the risk of general and cardiovascular death. This finding is of particular interest as patients with MASLD have disproportionately high cardiovascular mortality, exceeding liver-related death.

**Figure 14:**
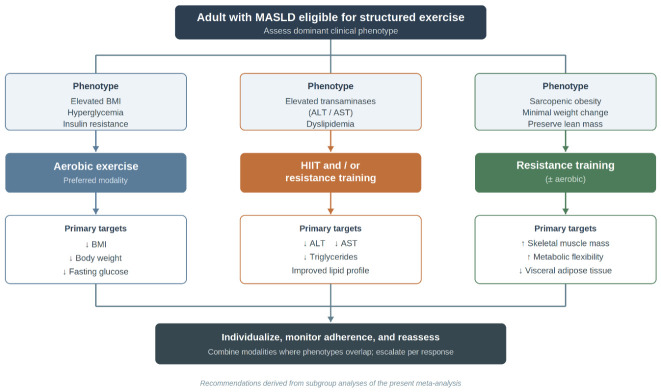
Evidence-based clinical algorithm for phenotype-guided exercise prescription across the MASLD spectrum. Modality assignments reflect the exercise type showing the greatest benefit for each outcome domain in the subgroup analyses of the present meta-analysis. The framework is intended to guide, not replace, individualized clinical judgment; combined or mixed modalities may be appropriate where phenotypes overlap. Abbreviations: MASLD, metabolic dysfunction–associated steatotic liver disease; BMI, body mass index; HIIT, high-intensity interval training; ALT, alanine aminotransferase; AST, aspartate aminotransferase.

Our subgroup analyses reveal that different exercise modalities produce distinct physiological benefits, suggesting that clinicians should tailor exercise prescriptions to specific patient goals. Aerobic exercise was the most effective modality for reducing BMI, body weight, and fasting blood glucose. This finding aligns with the well-established role of aerobic training in improving overall cardiovascular health and reducing mortality [41,59,60]. Conversely, high intensity training (HIIT) and resistance training were the primary contributors for improving lipid profiles and reducing liver enzymes (ALT and AST), as highlighted as well from literature [36,40]. Furthermore, resistance training holds a unique value in MASLD management because it enhances skeletal muscle mass and metabolic flexibility, even in the absence of significant weight loss [43,44]. These modality-specific effects are summarized as a practical, phenotype-guided prescription framework in Figure 14.

Overall, the review provides substantial evidence in favor of exercise as a treatment option for MASLD patients. However, some limitations warrant consideration. First, there was heterogeneity among included studies, driven by factors like exercise type, intensity, duration, and compliance. Such high variability reduces our confidence in the precision of the pooled estimates and suggests that the magnitude of exercise-induced benefits may not be uniform across all patient populations. Consequently, while the overall trend remains positive, these findings must be generalized to the clinical practice with caution as the optimal intensity and type of exercise likely require individualization based on specific patient phenotype and baseline metabolic health. This limits the ability to determine the best exercise strategy for MASLD. Second, the small sample size among the trials, along with short follow-up duration periods, hinders the long-term sustainability of exercise-based programs. Third, several outcomes were influenced by individual outlier studies, as shown by sensitivity analyses. Although the removal of these outliers improved the conclusions, it highlights the need for more standardized approaches in future studies. Furthermore, we acknowledge the limited scope and exploratory nature of our univariable meta-regression, which was restricted to investigating baseline BMI and age as potential sources of heterogeneity. These findings should not be over interpreted, and we caution against drawing definitive causal conclusions from these associations as they are intended to generate hypotheses rather than establish conclusive clinical drivers of treatment effect.

Future research on this subject should aim to conduct RCTs targeting standardized exercise programs, histological studies, and sensitive imaging to verify the effect of exercise on fibrosis progression. 

## 5. Conclusions

This meta-analysis and systematic review implies that exercise-based interventions are good sources of comprehensive and significant benefits among patients with MASLD. It not only lowers the BMI but also leads to improvement in liver enzymes, lipid profile, glycemic control, and reduces fibrosis scoring indices and quantitative measurements of liver fat, which may decrease the risk of cardiovascular death by increasing cardiovascular fitness. Importantly, these benefits occurred even in the absence of major weight loss, which underscores the fact that exercise improves MASLD through factors beyond simple weight reduction, including improved insulin sensitivity, reduced hepatic inflammation, and better metabolic flexibility.

## Figures and Tables

**Table 1:** Characteristics of the included studies.

Study	Population	Study Design	Locality	Type of Exercise	Duration of Exercise
Mucinski 2024 [17]	24 (I:16, C:8)	RCT	USA	High Intensity Interval Training	3 times/week for 10 months
Hallsworth 2011 [18]	19 (I:11, C:8)	RCT	UK	Resistance Exercise	8 weeks
Stine 2023 [42]	20 (I:12, C:8)	Post-Hoc Analysis	USA	Aerobic Exercise	5/week for 20 weeks
Kim 2025 [19]	66 (I:33, C:33)	RCT	Korea	Aerobic Exercise	30 min/day, 6 days/week for 12 weeks
Channapragada 2025 [20]	23 (I:15, C:8)	Post-Hoc Analysis	USA	Moderate Intensity Aerobic Exercise	5 sessions/week for 20 weeks
Cuthbertson 2024 [21]	88 (I:54, C:34)	Post-Hoc Analysis	UK, USA	Aerobic Exercise	12–20 weeks
Cuthbertson 2015 [22]	50 (I:30, C:20)	RCT	UK	Moderate Intensity Aerobic Exercise	3–5/week for 16 weeks
Harris 2023 [23]	23 (I:15, C:8)	Post-Hoc Analysis	USA	Moderate Intensity Aerobic Exercise	5 times/week for 20 weeks
Keating 2022 [24]	12 (I:7, C:5)	RCT	Australia	High Intensity Interval Training	3 days/week for 12 weeks
Hassabi 2023 [25]	40 (MI:13, HI:13, C:14)	RCT	Iran	Moderate and High Intensity Exercise	6 weeks
Stine 2022 [26]	28 (I:18, C:10)	RCT	USA	Moderate Intensity Exercise	5 sessions/week, each lasting 30 min for 20 weeks
Astinchap 2021 [27]	45 (ET:15, RT:15, C:15)	RCT	Iran	Endurance Training and Resistance Training	3 sessions/week for 8 weeks
Haufe 2021 [28]	314 (I:160, C:154)	RCT	Germany	Moderate Intensity Exercise	6 weeks
Kelardeh 2020 [29]	45 (I:12, C:11)	RCT	Iran	Non-linear Resistance Training	12 weeks
Whyte 2020 [30]	27 (I:15, C:12)	RCT	UK	Moderate Intensity Aerobic Exercise	16 weeks
Houghton 2017 [31]	24 (I:12, C:12)	RCT	UK	Cycling and Resistance Training	12 weeks
Shamsoddini 2015 [32]	30 (A:10, R:10, C:10)	RCT	Iran	Aerobic Exercise	8 weeks
Sullivan 2012 [33]	18 (I:12, C:6)	RCT	USA	Moderate Intensity Exercise	16 weeks
Achten 2003 [34]	9 (I:4, C:5)	RCT	UK	Walking or Cycling	12 weeks
Abdelbasset 2020 [35]	32 (I:16, C:16)	RCT	Egypt	Moderate and High Intensity Exercise	8 weeks
Hallsworth 2015 [36]	23 (I:11, C:12)	RCT	UK	High Intensity Exercise	12 weeks
Rajabi 2021 [37]	22 (I:11, C:11)	RCT	Iran	High Intensity Exercise	12 weeks
Reljic 2021 [41]	46 (I:29, C:17)	RCT	Germany	-	12 weeks
Norouzpour 2021 [38]	24 (I:12, C:12)	Quasi-Experimental Study	Iran	Aerobic Resistance Training	3 days/week for 10 weeks
Rezende 2016 [39]	40 (I:19, C:21)	RCT	Brazil	Aerobic Exercise	2 days/week for 24 weeks
Abdelbasset 2019 [40]	32 (I:16, C:16)	RCT	Egypt	High Intensity Interval Training	3 days/week for 8 weeks

**Table 2:** Baseline characteristics of included studies.

Study	Age, Years Mean (SD)		Gender (M/F)		BMI Mean (SD)		HbA1c Mean (SD)		VO_2_ peak, mL/kg/min Mean (SD)		Liver Fat MRI-PDFF % Mean (SD)	
	I	C	I	C	I	C	I	C	I	C	I	C
Mucinski 2024 [17]	47.2 (10)	47 (10.4)	7/9	2/6	40.7 (2.0)	37.5 (2.5)	7.2 (0.4)	7 (0.6)	-	-	-	-
Hallsworth 2011 [18]	52 (13.3)	62 (7.4)	-	-	32.3 (4.9)	32.3 (4.8)	6.1 (0.8)	6.5 (1.1)	21.8 (3.8)	18.5 (5.2)	14.0 (9.1)	11.2 (8.4)
Stine 2023 [42]	54.2 (5.47)	45.4 (6.3)	4/8	5/3	32.9 (1.99)	34.3 (3.2)	6.4 (0.5)	6.1 (0.6)	19.4 (2.4)	24.2 (3.2)	20.6 (2.08)	20 (6.5)
Kim 2025 [19]	75.2 (6.3)	75.5 (5.3)	13/20	13/20	27.6 (2.6)	26.6 (2.6)	-	-	24.6 (3.2)	24.4 (5.5)	-	-
Channapragada 2025 [20]	55.2 (10.6)	46.1 (11.0)	5/10	5/3	33.4 (4.9)	34.6 (5.3)	6.4 (1.3)	6.2 (1.1)	-	-	19.7 (5.8)	21.7 (11.6)
Cuthbertson 2024 [21]	51.8 (11.2)	50.9 (11.6)	34/20	25/9	33.3 (5.2)	33.3 (5.4)	-	-	23.4 (6.2)	23.5 (6.7)	25.0 (17.2)	22.8 (12.4)
Cuthbertson 2015 [22]	50.5 (2.94)	52.1 (3.48)	23/7	16/4	30.7 (0.9)	30 (1.5)	-	-	23.9 (1.5)	23.2 (1.3)	20.8 (5.3)	17.5 (6.1)
Harris 2023 [23]	55.2 (10.6)	46.1 (11.0)	5/10	5/3	33.4 (4.9)	34.6 (5.3)	6.4 (1.3)	6.2 (1.1)	20.6 (5.3)	24.6 (5.5)	19.7 (5.8)	21.7 (11.6)
Keating 2022 [24]	53.0 (12)	61 (5)	-	-	39.6 (7.1)	38.3 (6.9)	-	-	19.4 (7.0)	17.2 (8.2)	-	-
Hassabi 2023 [25]	MI = 53.20 (9.60) HI = 47.55 (9.65)	49.85 (11.27)	MI = 11/9 HI = 11/9	11/9	MI = 31.31 (4.74) HI = 30.70 (5.34)	30.67 (5.30)	MI = 5.82 (1.15) HI = 5.36 (1.01)	5.96 (1.47)	MI = 26.26 (6.22) HI = 31.72 (7.50)	26.75 (4.76)	-	-
Stine 2022 [26]	52.9 (11.5)	45.0 (10.2)	6/18	5/5	34.3 (4.9)	35.1 (4.9)	6.3 (1.2)	6.3 (1.2)	20.3 (5.1)	23.9 (5.3)	20.4 (7.7)	22.5 (10.5)
Astinchap 2021 [27]	-	-	-	-	ET = 30.1 (2.9) RT = 30.9 (3.6)	30.2 (3)	-	-	-	-	-	-
Haufe 2021 [28]	48.3 (7.9)	47.8 (8.5)	136/24	133/21	33.6 (5.3)	33 (5.4)	5.7 (1.0)	5.6 (0.9)	-	-	-	-
Kelardeh 2020 [29]	65.91 (3.31)	64.36 (2.97)	-	-	27.48 (1.43)	27.25 (1.34)	-	-	-	-	-	-
Whyte 2020 [30]	52.4 (7.5)	52.8 (10.3)	15/0	12/0	31.6 (3.2)	31.7 (3.6)	-	-	-	-	-	-
Houghton 2017 [31]	54 (12)	51 (16)	-	-	33 (7)	33 (5)	6.9 (1.3)	6.45 (1.0)	25 (8)	21 (5)	-	-
Shamsoddini 2015 [32]	A = 39.7 (6.3) R = 45.9 (7.3)	45.8 (7.3)	A = 10/0 R = 10/0	10/0	A = 28.1 (3.1) R = 30.6 (2.6)	28.2 (3.7)	-	-	-	-	-	-
Sullivan 2012 [33]	48.6 (2.2)	47.5 (3.1)	4/8	1/5	37.1 (1.1)	40.0 (2.2)	-	-	22.8 (1.3)	18.5 (2.9)	-	-
Achten 2003 [34]	42 (5)	43 (4)	9/0	9/0	32.8 (2.1)	32.3 (2.1)	-	-	-	-	-	-
Abdelbasset 2020 [35]	54.4 (5.8)	55.2 (4.3)	10/6	9/7	36.3 (4.5)	35.9 (5.3)	6.6 (0.4)	6.7 (0.6)	-	-	-	-
Hallsworth 2015 [36]	54 (10)	52 (12)	-	-	31 (4)	31 (5)	-	-	21.9 (6.2)	24.6 (5.7)	-	-
Rajabi 2021 [37]	42.09 (9.04)	43.82 (7.53)	0/11	0/11	30.77 (3.53)	32.03 (7.61)	-	-	-	-	-	-
Reljic 2021 [41]	-	-	-	-	40.9 (7.8)	39.4 (5.3)	-	-	-	-	-	-
Norouzpour 2021 [38]	56.10 (3.21)	56.25 (5.62)	0/12	0/12	-	-	-	-	-	-	-	-
Rezende 2016 [39]	56.2 (7.8)	54.5 (8.9)	0/19	0/21	34.1 (4.4)	32.0 (5.0)	6.7 (1.7)	6.3 (1.3)	19.8 (3.3)	22.1 (5.1)	-	-
Abdelbasset 2019 [40]	54.4 (5.8)	55.2 (4.3)	10/6	9/7	36.3 (4.5)	35.9 (5.3)	6.6 (0.4)	6.7 (0.6)	19.6 (2.6)	20.2 (2.3)	-	-

I: Intervention Group, C: Control Group, MI: Moderate Intensity Exercise, HI: High Intensity Exercise, ET: Endurance Training, RT: Resistance Training, A: Aerobic Exercise, R: Resistance Exercise.

## Data Availability

All the data is already available in the manuscript and the Supplementary File.

## Supplementary Materials

The following supporting information can be downloaded at: https://www.mdpi.com/article/10.3390/jcm15145737/s1, Figure S1: Risk-of-bias traffic light plot for the included randomized controlled trials assessed using RoB 2; Figure S2: Risk-of-bias summary plot for the included randomized controlled trials assessed using RoB 2; Figure S3: Risk-of-bias traffic light plot for the included non-randomized study assessed using ROBINS-I; Figure S4: Risk-of-bias summary plot for the included non-randomized study assessed using ROBINS-I; Figure S5: a: Leave-one-out sensitivity analysis for body mass index (BMI); b. Subgroup analysis based on exercise modality for body mass index (BMI): Aerobic = Aerobic Exercise, HIIT = High Intensity Interval Training, Resistance = Resistance Training, Mixed = A combination of any of the above three. Figure S6: a. Sensitivity analysis for weight, excluding non-randomized studies (Norouzpur 2021 [38]); b. Subgroup analysis based on exercise modality for weight; Aerobic = Aerobic Exercise, HIIT = High Intensity Interval Training, Resistance = Resistance Training; Figure S7: a. Leave-one-out sensitivity analysis for total cholesterol; b. Subgroup analysis based on exercise modality for total cholesterol; Aerobic = Aerobic Exercise, HIIT = High Intensity Interval Training, Resistance = Resistance Training; Figure S8: a. Leave-one-out sensitivity analysis for alanine aminotransferase (ALT) levels; b. Sensitivity analysis for alanine aminotransferase (ALT) levels, excluding non-randomized studies (Norouzpur 2021 [38]); c. Subgroup analysis based on exercise modality for alanine aminotransferase (ALT) levels; Aerobic = Aerobic Exercise, HIIT = High Intensity Interval Training, Resistance = Resistance Training; Figure S9: a. Leave-one-out sensitivity analysis for aspartate aminotransferase (AST) levels; b. Sensitivity analysis for aspartate aminotransferase (AST) levels, excluding non-randomized studies (Norouzpur 2021 [38]); c. Subgroup analysis based on exercise modality for aspartate aminotransferase (AST) levels; Aerobic = Aerobic Exercise, HIIT = High Intensity Interval Training, Resistance = Resistance Training; Figure S10: Leave-one-out sensitivity analysis for peak oxygen consumption (peak VO_2_); Figure S11: a. Leave-one-out sensitivity analysis for fasting blood glucose; b. Sensitivity analysis for fasting blood glucose, excluding non-randomized studies (Norouzpur 2021 [38]); c. Subgroup analysis based on exercise modality for fasting blood glucose; Aerobic = Aerobic Exercise, HIIT = High Intensity Interval Training, Resistance = Resistance Training, Mixed = A combination of any of the above three. Figure S12: Subgroup analysis based on exercise modality for HbA1c; Aerobic = Aerobic Exercise, HIIT = High Intensity Interval Training, Resistance = Resistance Training; Figure S13: a. Leave-one-out sensitivity analysis for homeostatic model assessment of insulin resistance (HOMA-IR); b. Sensitivity analysis for homeostatic model assessment of insulin resistance (HOMA-IR), excluding non-randomized studies (Norouzpur 2021 [38]); c. Subgroup analysis for homeostatic model assessment of insulin resistance (HOMA-IR); Aerobic = Aerobic Exercise, HIIT = High Intensity Interval Training, Resistance = Resistance Training, Mixed = A combination of any of the above three; Figure S14: Forest plot of the pooled mean difference in change in serum triglyceride levels. a. Leave-one-out sensitivity analysis for serum triglyceride levels. b. Sensitivity analysis for serum triglyceride levels, excluding non-randomized studies (Norouzpur 2021 [38]). c. Subgroup analysis based on exercise modality for serum triglyceride levels; Aerobic = Aerobic Exercise, HIIT = High Intensity Interval Training, Resistance = Resistance Training, Mixed = A combination of any of the above three.; Figure S15: Forest plot of the pooled mean difference in change in high-density lipoprotein (HDL) levels. a. Leave-one-out sensitivity analysis for high-density lipoprotein (HDL) levels; Figure S16: Forest plot of the pooled mean difference in change in low-density lipoprotein (LDL) levels. a. Leave-one-out sensitivity analysis for low-density lipoprotein (LDL) levels.; Figure S17: Forest plot of the pooled mean difference in change in intrahepatic triglyceride levels. a. Leave-one-out sensitivity analysis for intrahepatic triglyceride levels; Figure S18: Forest plot of the pooled mean difference in change in magnetic resonance imaging–proton density fat fraction (MRI-PDFF). Figure S19: Forest plot of the pooled risk ratio for achieving greater than 30% reduction in MRI-PDFF. Table S1: PRISMA Checklist for our study.

## Abbreviations

MASLD	metabolic dysfunction-associated steatotic liver disease
NAFLD	nonalcoholic fatty liver disease
RCT	randomized controlled trial
BMI	body mass index
ALT	alanine aminotransferase
AST	aspartate aminotransferase
HDL	high-density lipoprotein
LDL	low-density lipoprotein
FIB-4	fibrosis-4 index
NFS	NAFLD fibrosis score
HOMA-IR	homeostatic model assessment of insulin resistance
MRI-PDFF	magnetic resonance imaging–proton density fat fraction
