# Supplementary material for: Exercise Versus Standard Therapy in Steatotic Liver Disease Spectrum: A Systematic Review, Meta-Analysis, and Meta-Regression
Source: J Clin Med. 2026 Jul 22;15(14):5737. doi: 10.3390/jcm15145737 (PMC13412719; doi:10.3390/jcm15145737)
Supplement: Supplementary file 1 [file jcm-15-05737-s001.zip › jcm-4406182-supplementary.pdf]

Supplementary File

Search strategy:

Pubmed	(((Non-alcoholic Fatty Liver Disease OR Non-alcoholic Steatohepatitis OR Fatty Liver OR MASH OR MASLD)) AND ((Exercise OR Exercise Therapy OR Physical Fitness OR Aerobic exercise OR Exercise training OR Physical activity))) AND ((Liver Function Tests OR Biomarkers OR Magnetic Resonance Imaging OR Liver biopsy OR Alanine Transaminase OR Aspartate Aminotransferases OR MRI-PDFF OR MASH Resolution Index))
Cochrane	(((Non-alcoholic Fatty Liver Disease OR Non-alcoholic Steatohepatitis OR Fatty Liver OR MASH OR MASLD)) AND ((Exercise OR Exercise Therapy OR Physical Fitness OR Aerobic exercise OR Exercise training OR Physical activity))) AND ((Liver Function Tests OR Biomarkers OR Magnetic Resonance Imaging OR Liver biopsy OR Alanine Transaminase OR Aspartate Aminotransferases OR MRI-PDFF OR MASH Resolution Index))
clinicaltrials.gov	(((Non-alcoholic Fatty Liver Disease OR Non-alcoholic Steatohepatitis OR Fatty Liver OR MASH OR MASLD)) AND ((Exercise OR Exercise Therapy OR Physical Fitness OR Aerobic exercise OR Exercise training OR Physical activity))) AND ((Liver Function Tests OR Biomarkers OR Magnetic

	Resonance Imaging OR Liver biopsy OR Alanine Transaminase OR Aspartate Aminotransferases OR MRI-PDFF OR MASH Resolution Index))
Scopus	TITLE-ABS-KEY("non-alcoholic fatty liver disease" OR "nonalcoholic fatty liver disease" OR NAFLD OR "non-alcoholic steatohepatitis" OR "nonalcoholic steatohepatitis" OR NASH OR "fatty liver" OR MASH OR MASLD)<br>AND<br>TITLE-ABS-KEY(exercise OR "exercise therapy" OR "physical fitness" OR "aerobic exercise" OR "exercise training" OR "physical activity")<br>AND<br>TITLE-ABS-KEY("liver function tests" OR biomarker OR biomarkers OR "magnetic resonance imaging" OR "MRI PDFF" OR "MRI-PDFF" OR "liver biopsy" OR "alanine transaminase" OR "aspartate aminotransferase" OR ALT OR AST OR "MASH Resolution Index")

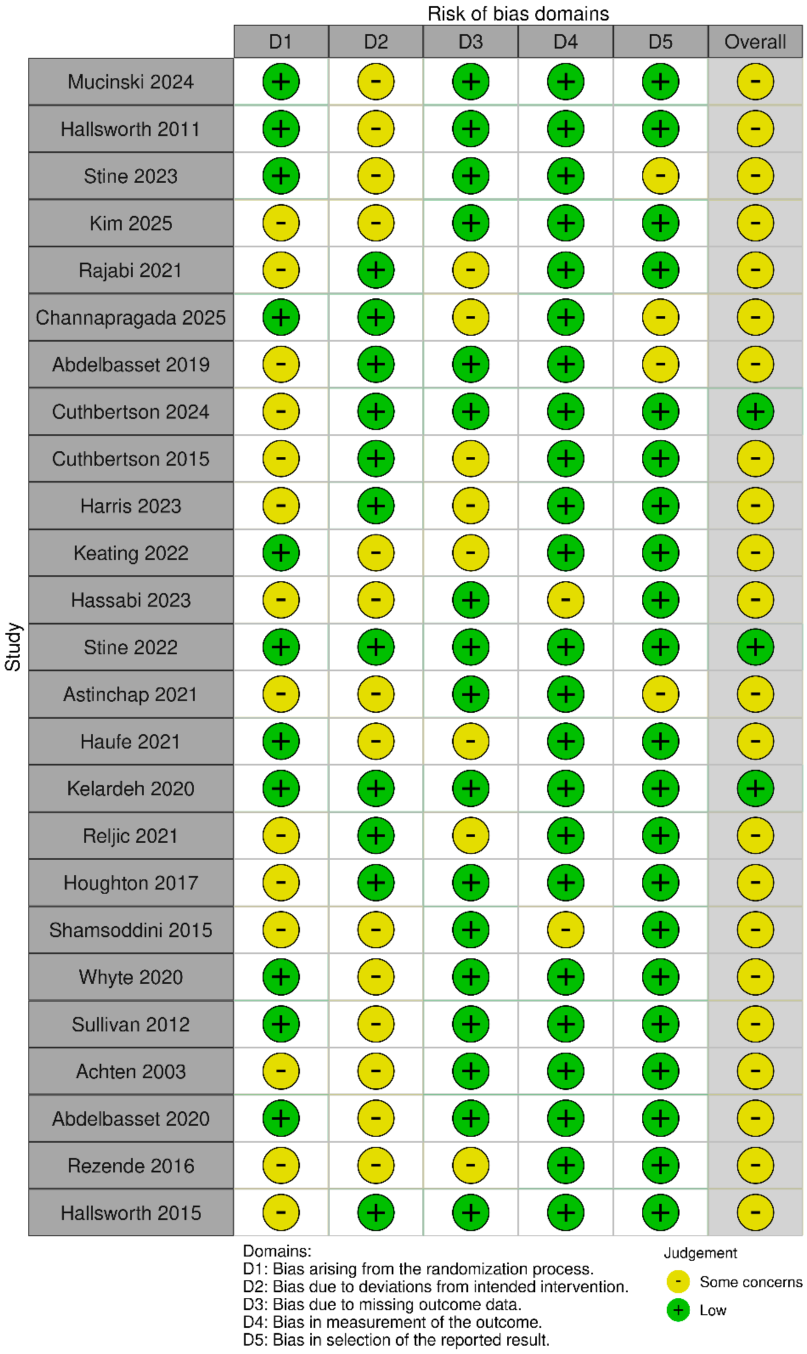

Supplementary Figure S1: Risk-of-bias traffic light plot for the included randomized controlled trials assessed using RoB 2.

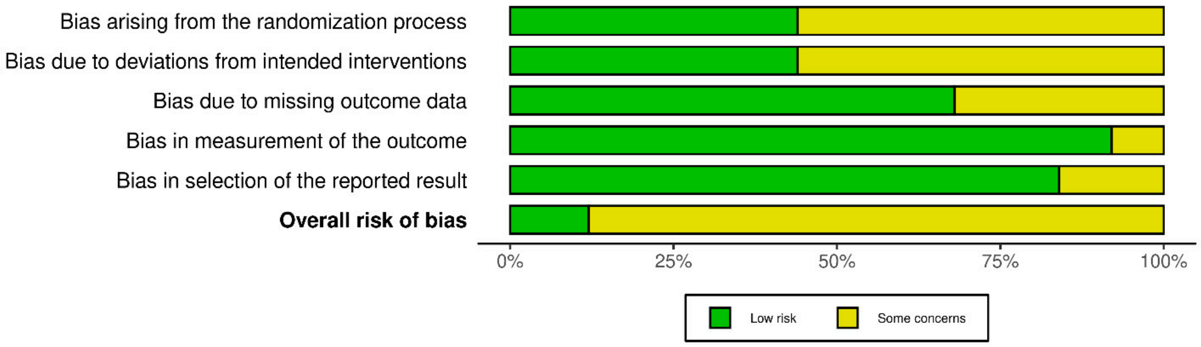

Supplementary Figure S2: Risk-of-bias summary plot for the included randomized controlled trials assessed using RoB 2.

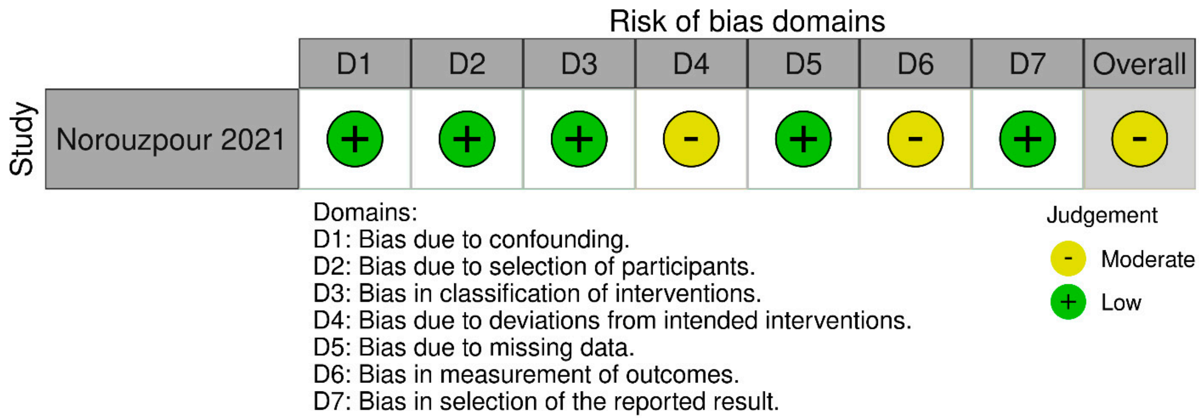

Supplementary Figure S3. Risk-of-bias traffic light plot for the included non-randomized study assessed using ROBINS-I.

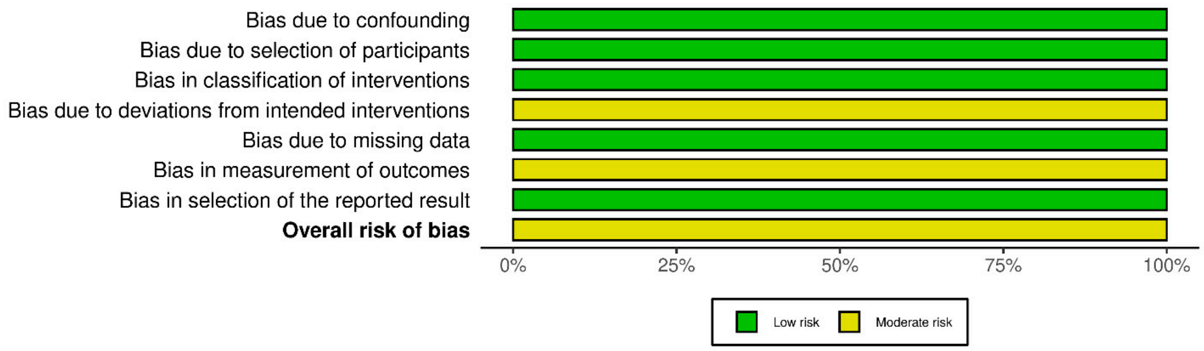

Supplementary Figure S4. Risk-of-bias summary plot for the included non-randomized study assessed using ROBINS-I.

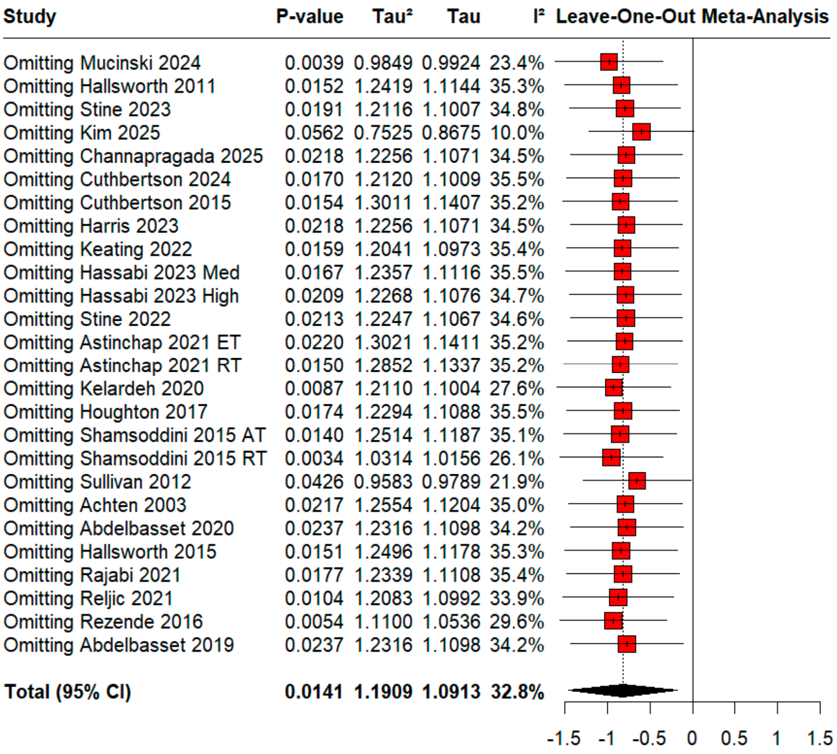

Supplementary Figure S5a. Leave-one-out sensitivity analysis for body mass index (BMI).

Study	Exercise Total	Exercise Mean	Exercise SD	Control Total	Control Mean	Control SD	MD	95%-CI	Weight
**subgroup = Aerobic**									
Stine 2023	12	32.70	5.6000	8	35.20	6.8000	-2.50	[-8.18; 3.18]	1.5%
Kim 2025	33	24.70	3.0000	33	27.80	3.2000	-3.10	[-4.60; -1.60]	7.8%
Channapragada 2025	15	32.80	0.3000	8	35.00	5.4000	-2.20	[-5.95; 1.55]	2.9%
Cuthbertson 2024	54	34.10	17.7833	34	35.20	14.5774	-1.10	[-7.92; 5.72]	1.0%
Cuthbertson 2015	30	29.96	3.1900	20	30.33	3.9900	-0.37	[-2.46; 1.72]	6.0%
Harris 2023	15	32.80	0.3000	8	35.00	5.4000	-2.20	[-5.95; 1.55]	2.9%
Hassabi 2023 Med	13	30.56	5.7100	14	31.22	6.1000	-0.66	[-5.11; 3.79]	2.2%
Hassabi 2023 High	13	29.12	4.5500	14	31.22	6.1000	-2.10	[-6.14; 1.94]	2.6%
Stine 2022	18	32.80	4.5000	10	35.00	5.4000	-2.20	[-6.14; 1.74]	2.7%
Astinchap 2021 ET	15	29.30	2.9000	15	30.50	3.0000	-1.20	[-3.31; 0.91]	5.9%
Shamsoddini 2015 AT	10	27.50	3.5000	10	27.50	3.9000	0.00	[-3.25; 3.25]	3.5%
Sullivan 2012	12	37.10	1.1000	6	40.10	2.1000	-3.00	[-4.79; -1.21]	6.9%
Achten 2003	4	30.70	1.4000	5	32.30	3.1000	-1.60	[-4.64; 1.44]	3.9%
Rezende 2016	19	33.61	4.5800	21	31.82	4.7900	1.79	[-1.11; 4.69]	4.1%
**Random effects model**	**263**			**206**			**-1.59**	**[-2.42; -0.75]**	**53.7%**
Heterogeneity: $I^2 = 9\%$, $\tau^2 = 0.7545$, $\chi^2_{13} = 14.28$ ($p = 0.3541$)									
**subgroup = HIIT**									
Abdelbasset 2019	16	34.10	3.1000	16	36.20	5.5000	-2.10	[-5.19; 0.99]	3.8%
Mucinski 2024	16	37.50	1.7000	8	36.30	2.1000	1.20	[-0.48; 2.88]	7.2%
Keating 2022	7	39.00	7.6000	5	39.20	7.7000	-0.20	[-8.99; 8.59]	0.7%
Abdelbasset 2020	16	34.10	3.1000	16	36.20	5.5000	-2.10	[-5.19; 0.99]	3.8%
Hallsworth 2015	11	31.00	4.1000	12	31.20	4.6000	-0.20	[-3.76; 3.36]	3.1%
Reljic 2021	29	39.60	7.8000	17	38.50	5.6000	1.10	[-2.79; 4.99]	2.7%
**Random effects model**	**95**			**74**			**-0.22**	**[-1.91; 1.47]**	**21.2%**
Heterogeneity: $I^2 = 14.1\%$, $\tau^2 = 1.0815$, $\chi^2_5 = 5.82$ ($p = 0.3242$)									
**subgroup = Resistance**									
Hallsworth 2011	11	32.30	4.5000	8	32.50	4.2000	-0.20	[-4.14; 3.74]	2.7%
Astinchap 2021 ST	15	30.20	3.7000	15	30.50	3.0000	-0.30	[-2.71; 2.11]	5.1%
Kelardeh 2020	12	27.46	1.4500	11	27.21	1.2900	0.25	[-0.87; 1.37]	9.2%
Houghton 2017	12	33.00	7.0000	12	34.00	5.0000	-1.00	[-5.87; 3.87]	1.9%
Shamsoddini 2015 RT	10	29.90	2.6000	10	27.50	3.9000	2.40	[-0.51; 5.31]	4.1%
**Random effects model**	**60**			**56**			**0.32**	**[-0.73; 1.36]**	**22.9%**
Heterogeneity: $I^2 = 0\%$, $\tau^2 = 0$, $\chi^2_4 = 2.59$ ($p = 0.6290$)									
**subgroup = Mixed**									
Rajabi 2021	11	30.37	3.7100	11	31.41	6.7400	-1.04	[-5.59; 3.51]	2.1%
**Random effects model**	**429**			**347**			**-0.82**	**[-1.46; -0.18]**	**100.0%**
Heterogeneity: $I^2 = 32.8\%$, $\tau^2 = 1.1909$, $\chi^2_{25} = 37.19$ ($p = 0.0554$)									
Test for overall effect: $t_{25} = -2.64$ ($p = 0.0141$)									
Test for subgroup differences: $\chi^2_3 = 12.74$, df = 3 ($p = 0.0052$)									

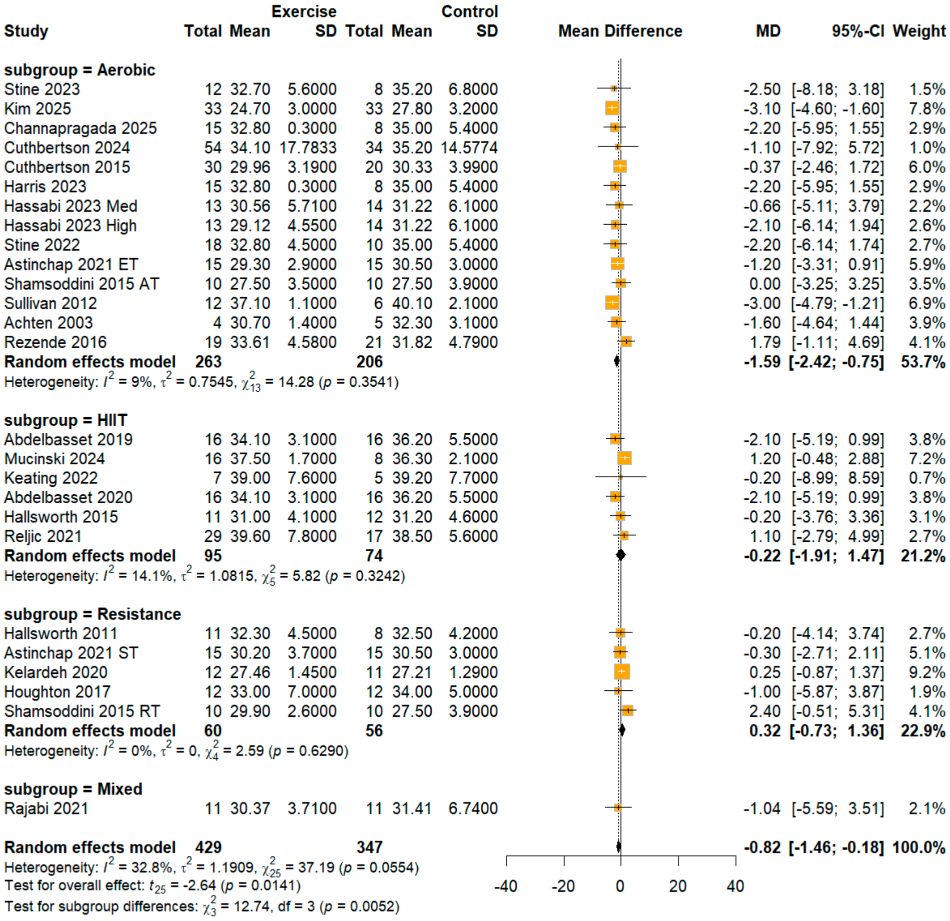

Supplementary Figure S5b. Subgroup analysis based on exercise modality for body mass index (BMI): Aerobic = Aerobic Exercise, HIIT=High Intensity Interval Training, Resistance=Resistance Training, Mixed=A combination of any of the above three.

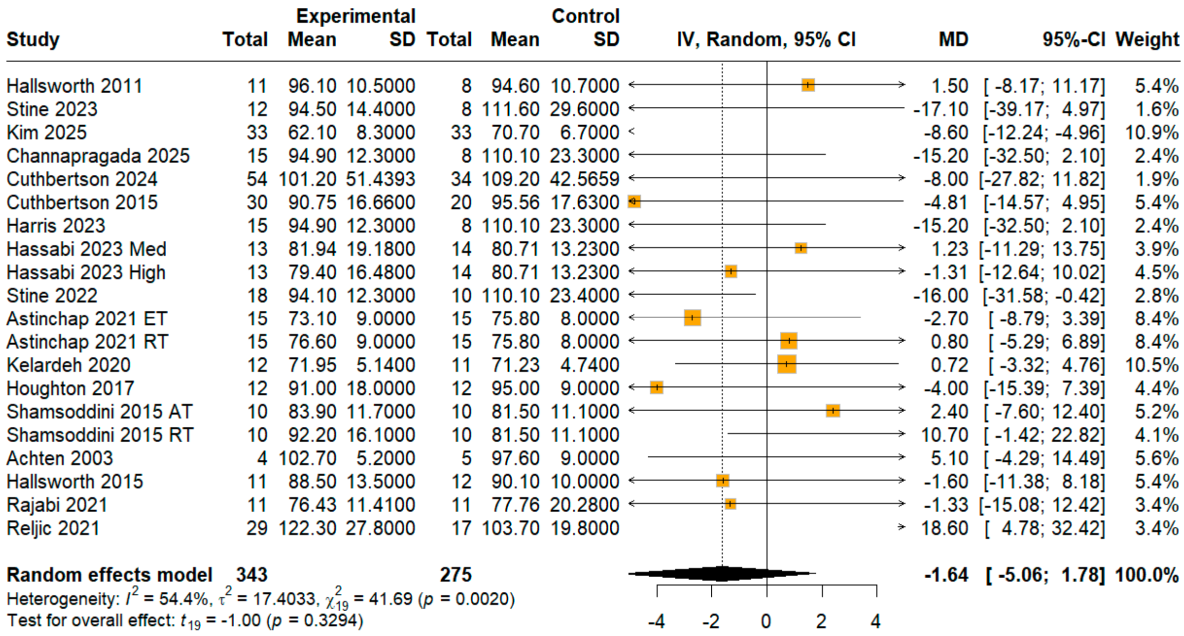

Supplementary Figure S6a. Sensitivity analysis for weight, excluding non-randomized studies (Norouzpur 2021).

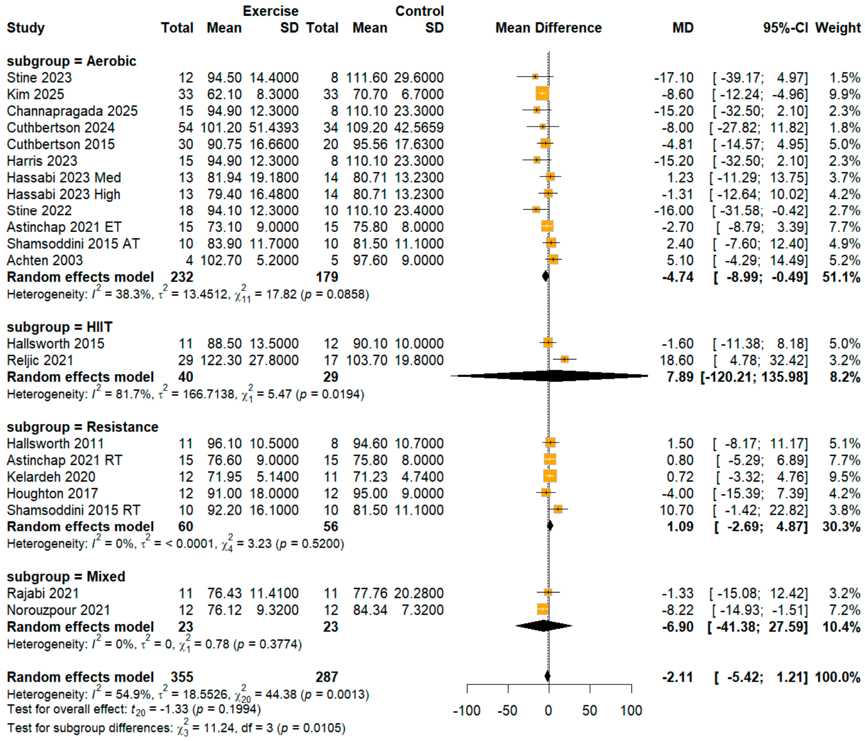

Supplementary Figure S6b. Subgroup analysis based on exercise modality for weight; Aerobic=Aerobic Exercise, HIIT=High Intensity Interval Training, Resistance=Resistance Training.

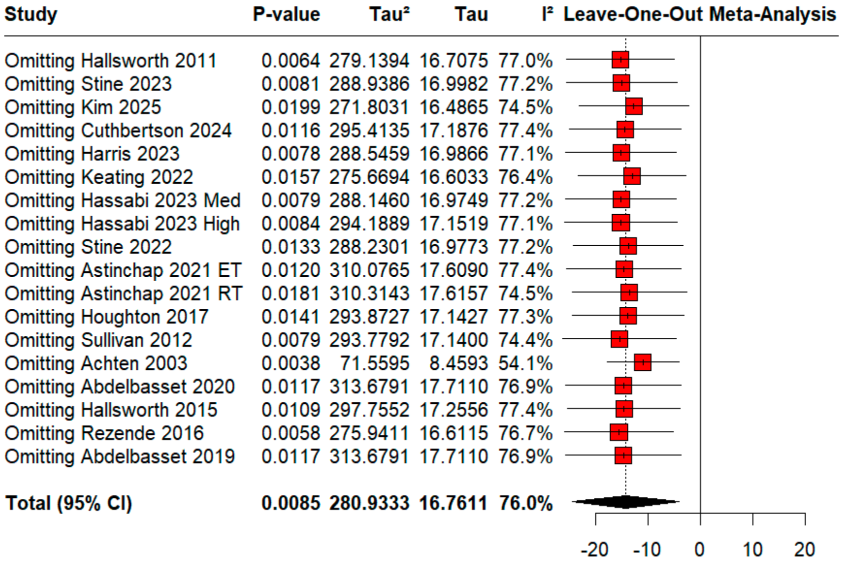

Supplementary Figure S7a. Leave-one-out sensitivity analysis for total cholesterol.

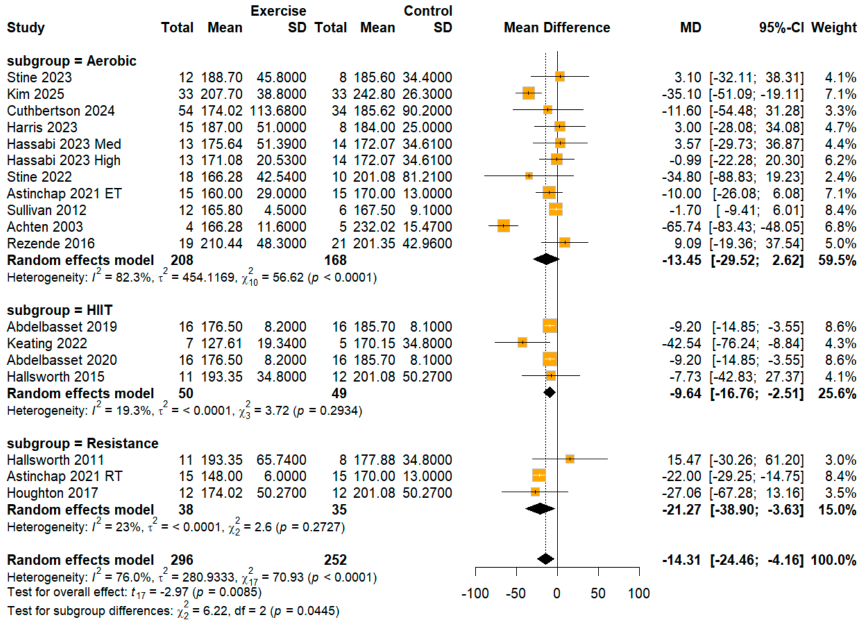

Supplementary Figure S7b. Subgroup analysis based on exercise modality for total cholesterol;

Aerobic=Aerobic Exercise, HIIT=High Intensity Interval Training, Resistance=Resistance Training.

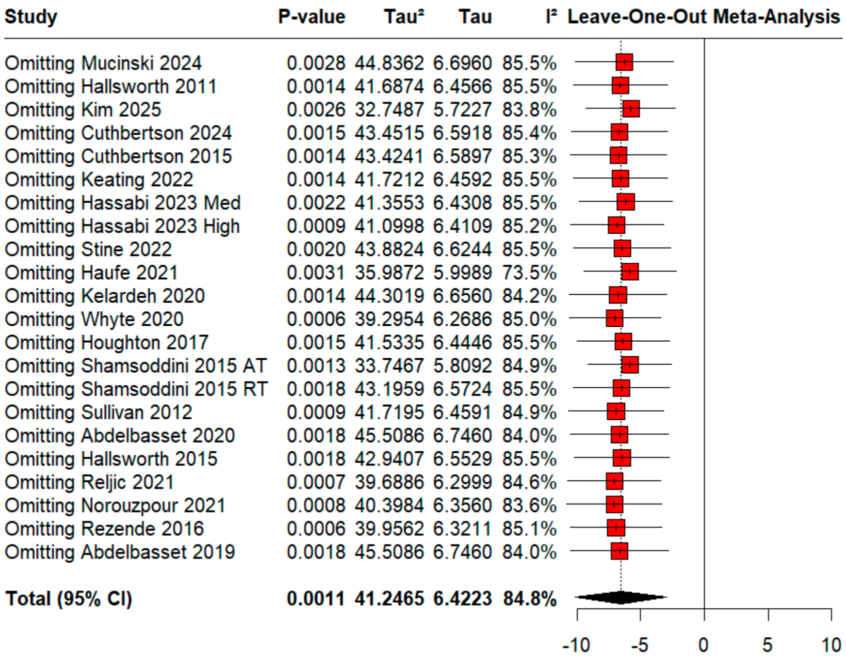

Supplementary Figure S8a. Leave-one-out sensitivity analysis for alanine aminotransferase (ALT) levels.

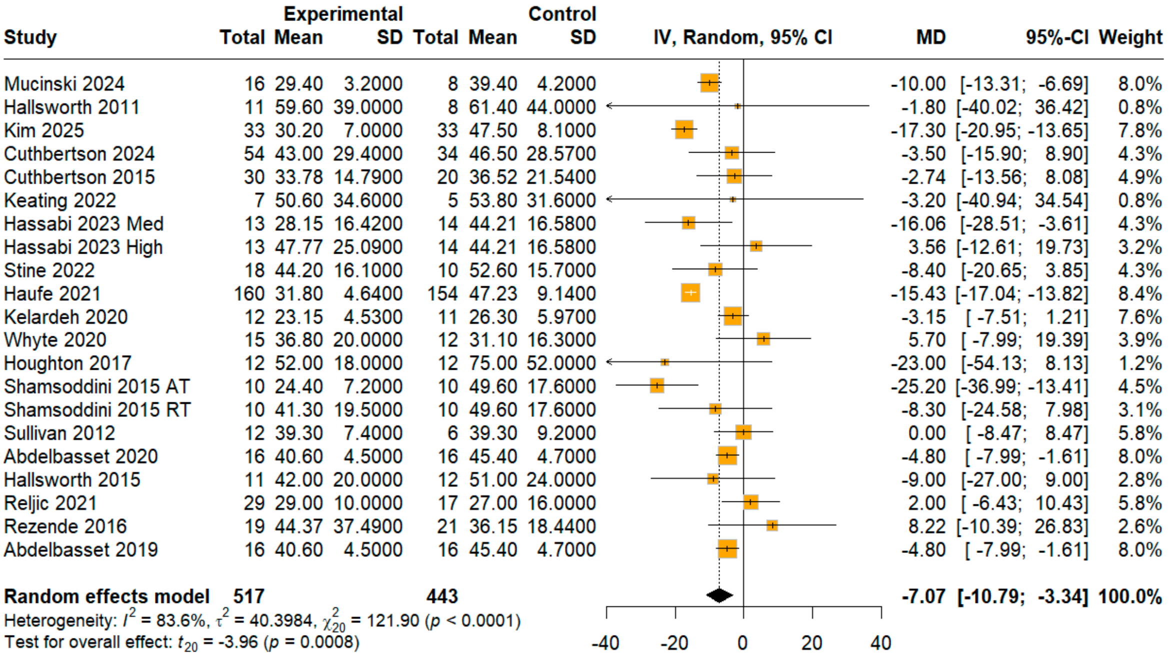

Supplementary Figure S8b. Sensitivity analysis for alanine aminotransferase (ALT) levels, excluding non-randomized studies (Norouzpur 2021).

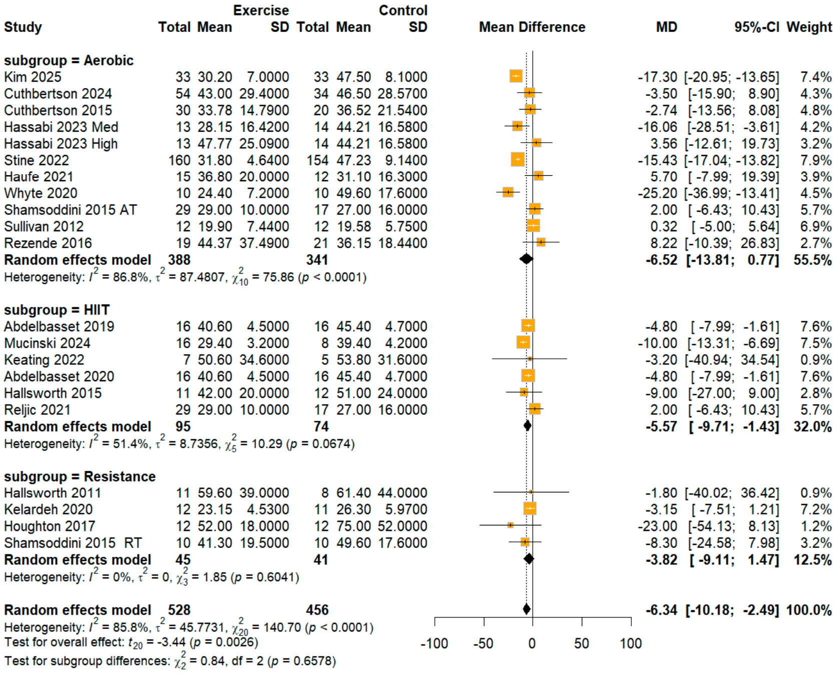

Supplementary Figure S8c. Subgroup analysis based on exercise modality for alanine aminotransferase (ALT) levels; Aerobic=Aerobic Exercise, HIIT=High Intensity Interval Training, Resistance=Resistance Training.

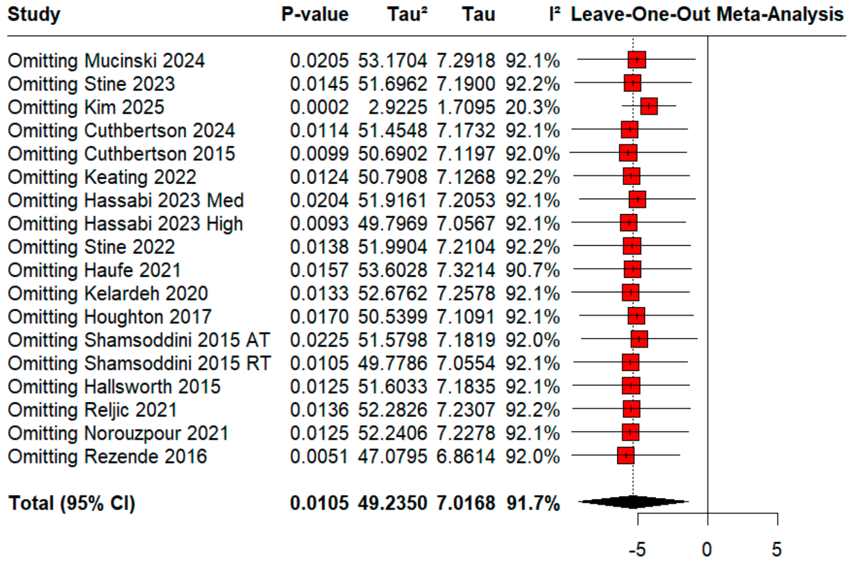

Supplementary Figure S9a. Leave-one-out sensitivity analysis for aspartate aminotransferase (AST) levels.

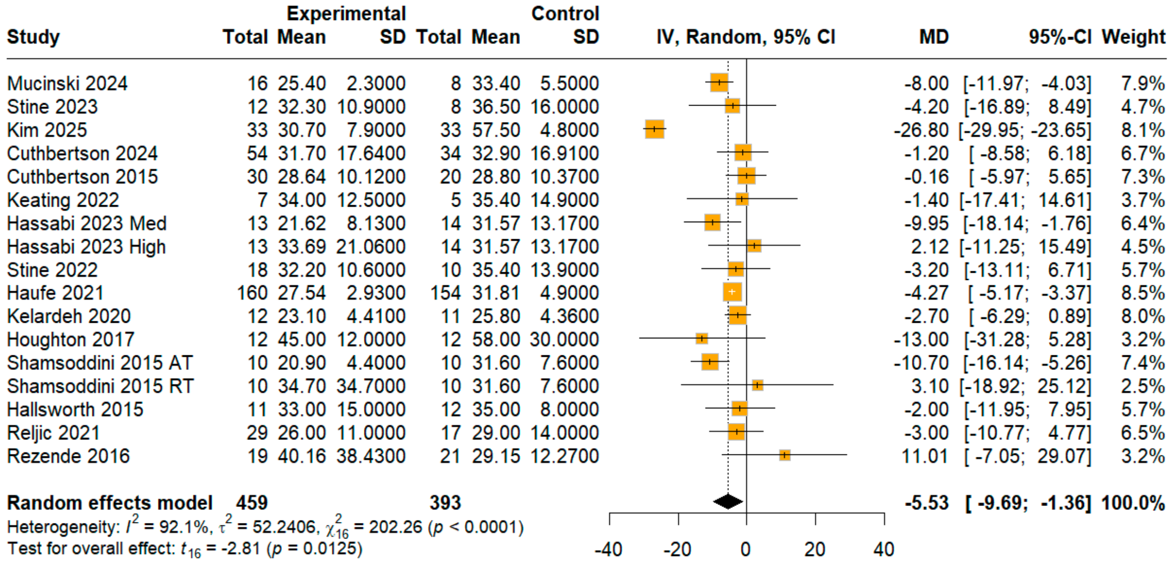

Supplementary Figure S9b. Sensitivity analysis for aspartate aminotransferase (AST) levels, excluding non-randomized studies (Norouzpur 2021).

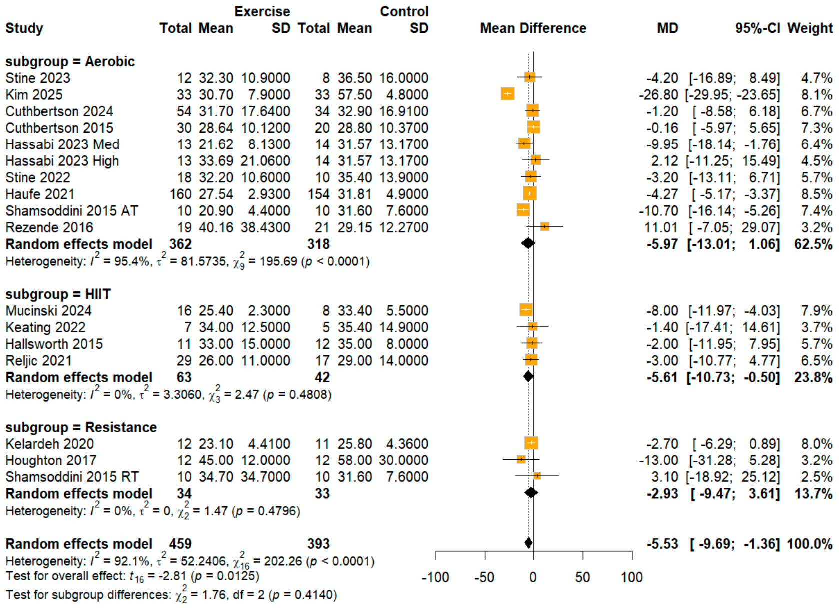

Supplementary Figure S9c. Subgroup analysis based on exercise modality for aspartate aminotransferase (AST) levels; Aerobic=Aerobic Exercise, HIIT=High Intensity Interval Training, Resistance=Resistance Training.

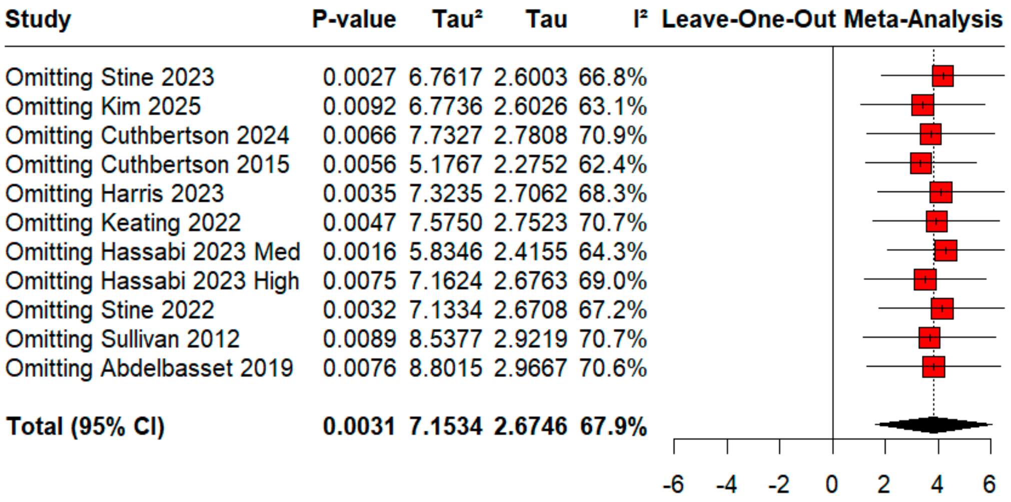

Supplementary Figure S10. Leave-one-out sensitivity analysis for peak oxygen consumption (peak VO_2).

Study	P-value	Tau²	Tau	I²
Omitting Hallsworth 2011	0.0101	130.5382	11.4253	86.3%
Omitting Stine 2023	0.0123	129.0706	11.3609	86.3%
Omitting Kim 2025	0.0188	57.1031	7.5567	59.3%
Omitting Cuthbertson 2024	0.0094	132.4084	11.5069	86.4%
Omitting Cuthbertson 2015	0.0067	136.0972	11.6661	84.9%
Omitting Harris 2023	0.0091	128.5423	11.3376	86.3%
Omitting Keating 2022	0.0075	131.2995	11.4586	86.3%
Omitting Hassabi 2023 Med	0.0064	134.8312	11.6117	85.9%
Omitting Hassabi 2023 High	0.0099	139.9485	11.8300	86.3%
Omitting Stine 2022	0.0092	128.3481	11.3291	86.3%
Omitting Astinchap 2021 ET	0.0115	137.9535	11.7454	86.4%
Omitting Astinchap 2021 RT	0.0147	132.8451	11.5258	86.3%
Omitting Whyte 2020	0.0036	122.7375	11.0787	83.2%
Omitting Houghton 2017	0.0028	121.8683	11.0394	85.8%
Omitting Achten 2003	0.0164	133.9012	11.5716	86.0%
Omitting Hallsworth 2015	0.0054	130.4353	11.4208	86.1%
Omitting Rajabi 2021	0.0035	122.5207	11.0689	84.8%
Omitting Norouzpour 2021	0.0081	130.3519	11.4172	86.3%
Omitting Abdelbasset 2019	0.0119	135.9970	11.6618	86.4%
**Total (95% CI)**	**0.0067**	**128.0408**	**11.3155**	**85.6%**

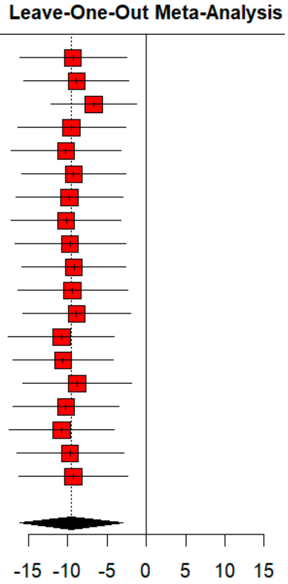

Supplementary Figure S11a. Leave-one-out sensitivity analysis for fasting blood glucose.

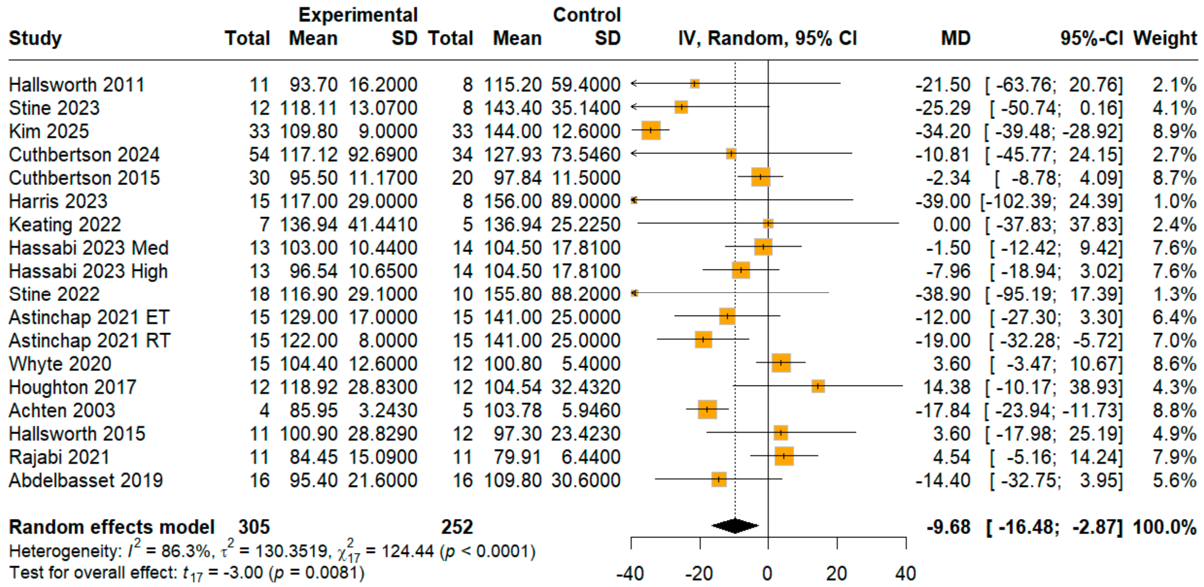

Supplementary Figure S11b. Sensitivity analysis for fasting blood glucose, excluding non-randomized studies (Norouzpur 2021).

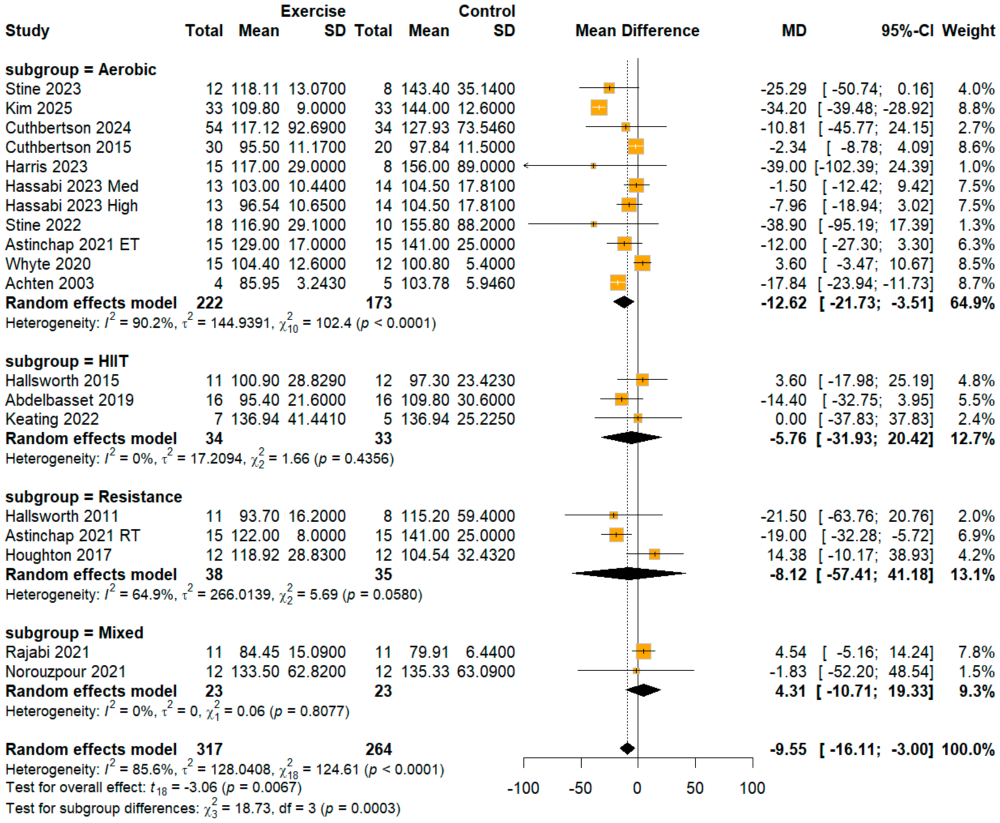

Supplementary Figure S11c. Subgroup analysis based on exercise modality for fasting blood glucose; Aerobic=Aerobic Exercise, HIIT=High Intensity Interval Training, Resistance=Resistance Training, Mixed=A combination of any of the above three.

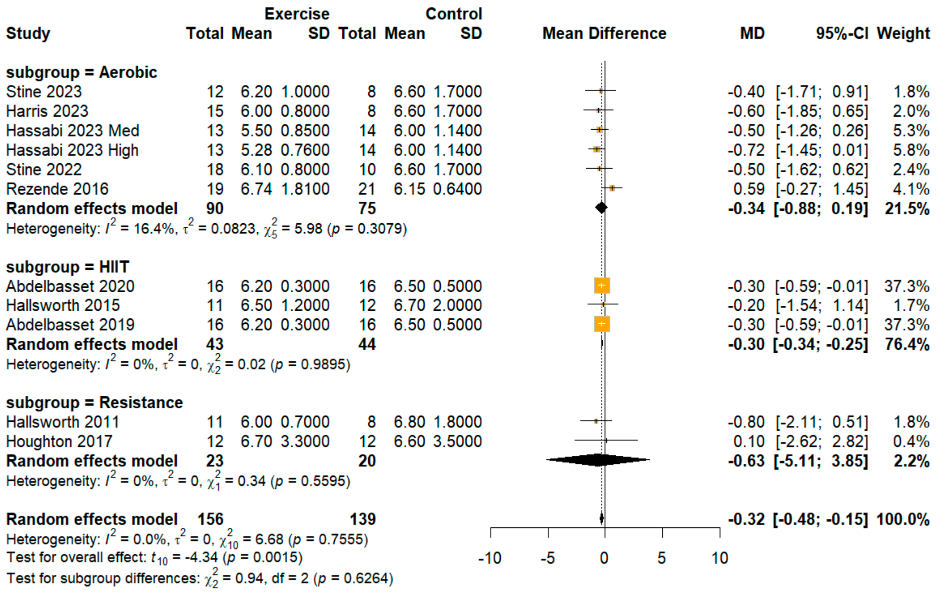

Supplementary Figure S12. Subgroup analysis based on exercise modality for HbA1c; Aerobic=Aerobic Exercise, HIIT=High Intensity Interval Training, Resistance=Resistance Training.

Study	P-value	Tau^2	Tau	I^2
Omitting Hallsworth 2011	0.1066	0.9141	0.9561	71.7%
Omitting Cuthbertson 2024	0.1108	1.0054	1.0027	71.7%
Omitting Cuthbertson 2015	0.1195	1.0484	1.0239	70.9%
Omitting Harris 2023	0.1038	0.8700	0.9328	71.5%
Omitting Stine 2022	0.1038	0.8707	0.9331	71.4%
Omitting Astinchap 2021 ET	0.0266	0.0278	0.1666	8.4%
Omitting Astinchap 2021 RT	0.1356	0.9842	0.9921	71.6%
Omitting Houghton 2017	0.0654	0.8983	0.9478	68.0%
Omitting Abdelbasset 2020	0.1450	1.0129	1.0064	71.4%
Omitting Rajabi 2021	0.0289	0.7202	0.8487	67.9%
Omitting Norouzpour 2021	0.1140	0.9378	0.9684	71.7%
Omitting Rezende 2016	0.0502	0.8275	0.9097	69.8%
Omitting Abdelbasset 2019	0.1450	1.0129	1.0064	71.4%
**Total (95% CI)**	**0.0864**	**0.8645**	**0.9298**	**69.2%**

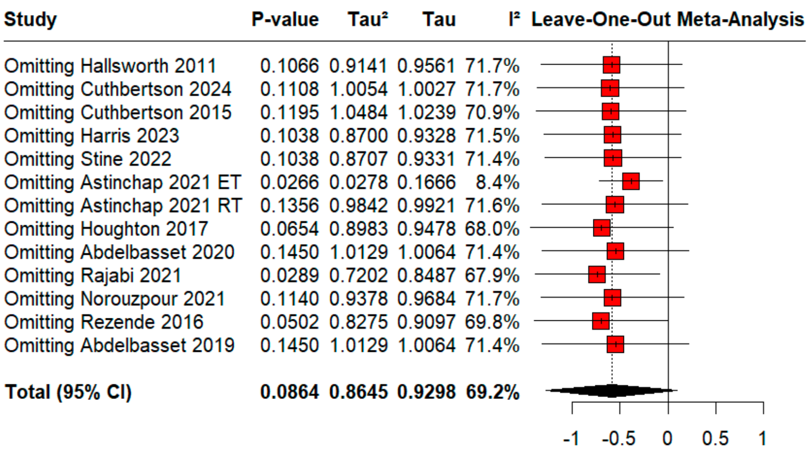

Supplementary Figure S13a. Leave-one-out sensitivity analysis for homeostatic model assessment of insulin resistance (HOMA-IR).

Study	Experimental Total	Experimental Mean	Experimental SD	Control Total	Control Mean	Control SD	MD	95%-CI	Weight
Hallsworth 2011	11	4.60	4.6000	8	5.10	2.5000	-0.50	[-3.72; 2.72]	3.7%
Cuthbertson 2024	54	1.80	2.9400	34	2.20	2.3300	-0.40	[-1.51; 0.71]	10.6%
Cuthbertson 2015	30	2.10	0.3900	20	2.57	0.5100	-0.47	[-0.73; -0.21]	13.9%
Harris 2023	15	9.20	6.5000	8	12.60	14.9000	-3.40	[-14.24; 7.44]	0.4%
Stine 2022	18	8.80	6.5000	10	12.60	14.9000	-3.80	[-13.51; 5.91]	0.5%
Astinchap 2021 ET	15	2.20	0.6000	15	5.10	1.7000	-2.90	[-3.81; -1.99]	11.5%
Astinchap 2021 RT	15	4.20	2.3000	15	5.10	1.7000	-0.90	[-2.35; 0.55]	9.0%
Houghton 2017	12	1.90	0.8000	12	1.70	1.0000	0.20	[-0.52; 0.92]	12.4%
Abdelbasset 2020	16	4.10	0.6000	16	4.98	1.8000	-0.88	[-1.81; 0.05]	11.4%
Rajabi 2021	11	3.62	2.7900	11	2.24	1.0600	1.38	[-0.38; 3.14]	7.6%
Rezende 2016	19	3.59	3.7900	21	2.74	1.1800	0.85	[-0.93; 2.63]	7.6%
Abdelbasset 2019	16	4.10	0.6000	16	4.98	1.8000	-0.88	[-1.81; 0.05]	11.4%
**Random effects model**	**232**			**186**			**-0.58**	**[-1.33; 0.16]**	**100.0%**

Heterogeneity: $I^2 = 71.7\%$, $\tau^2 = 0.9378$, $\chi^2_{11} = 38.90$ ($p < 0.0001$)
Test for overall effect: $t_{11} = -1.72$ ($p = 0.1140$)

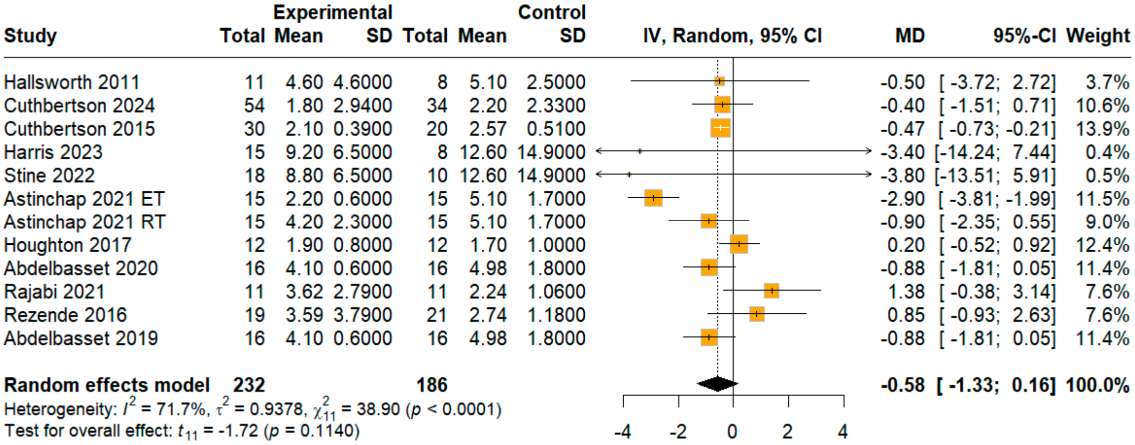

Supplementary Figure S13b. Sensitivity analysis for homeostatic model assessment of insulin resistance (HOMA-IR), excluding non-randomized studies (Norouzpur 2021).

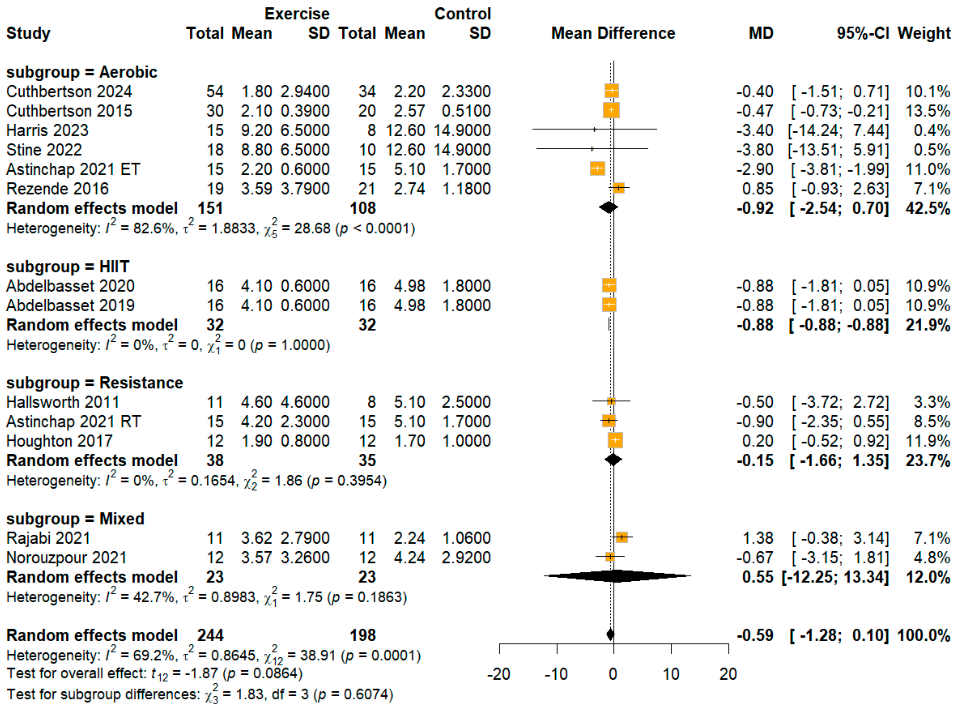

Supplementary Figure S13c. Subgroup analysis for homeostatic model assessment of insulin resistance (HOMA-IR); Aerobic=Aerobic Exercise, HIIT=High Intensity Interval Training, Resistance=Resistance Training, Mixed=A combination of any of the above three.

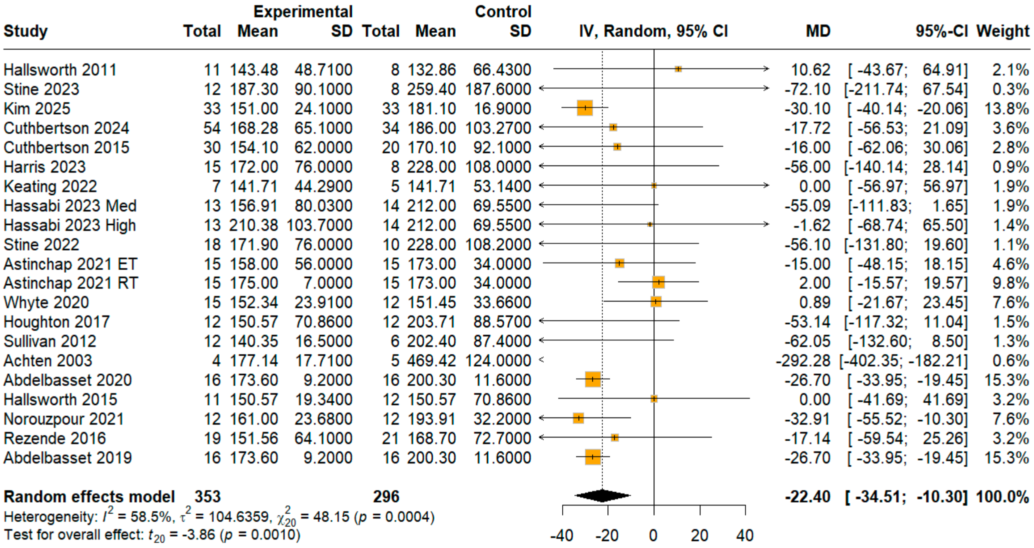

Supplementary Figure S14. Forest plot of the pooled mean difference in change in serum triglyceride levels.

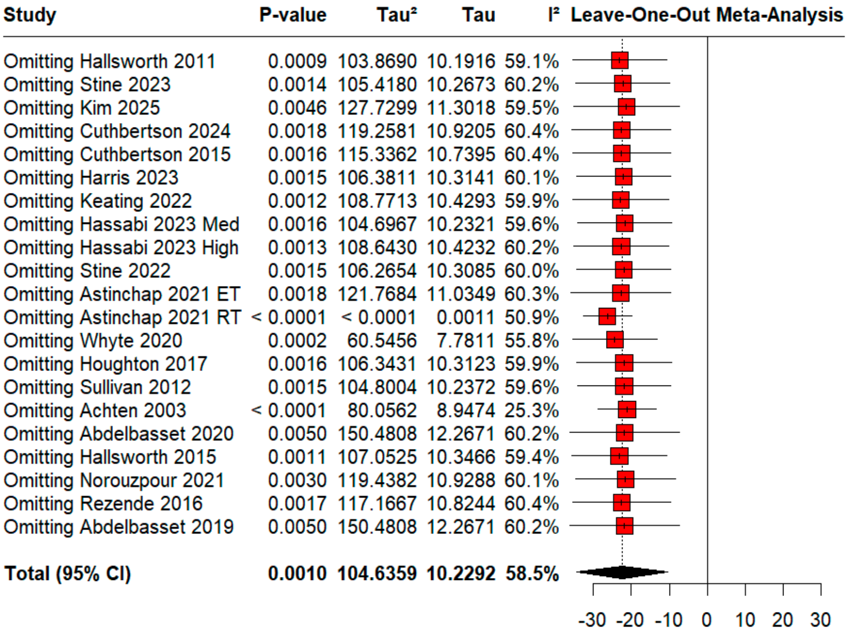

Supplementary Figure S14a. Leave-one-out sensitivity analysis for serum triglyceride levels.

Study	Experimental Total	Experimental Mean	Experimental SD	Control Total	Control Mean	Control SD	IV, Random, 95% CI	MD	95%-CI	Weight
Hallsworth 2011	11	143.48	48.7100	8	132.86	66.4300		10.62	[-43.67; 64.91]	2.4%
Stine 2023	12	187.30	90.1000	8	259.40	187.6000		-72.10	[-211.74; 67.54]	0.4%
Kim 2025	33	151.00	24.1000	33	181.10	16.9000		-30.10	[-40.14; -20.06]	14.5%
Cuthbertson 2024	54	168.28	65.1000	34	186.00	103.2700		-17.72	[-56.53; 21.09]	4.1%
Cuthbertson 2015	30	154.10	62.0000	20	170.10	92.1000		-16.00	[-62.06; 30.06]	3.1%
Harris 2023	15	172.00	76.0000	8	228.00	108.0000		-56.00	[-140.14; 28.14]	1.1%
Keating 2022	7	141.71	44.2900	5	141.71	53.1400		0.00	[-56.97; 56.97]	2.2%
Hassabi 2023 Med	13	156.91	80.0300	14	212.00	69.5500		-55.09	[-111.83; 1.65]	2.2%
Hassabi 2023 High	13	210.38	103.7000	14	212.00	69.5500		-1.62	[-68.74; 65.50]	1.6%
Stine 2022	18	171.90	76.0000	10	228.00	108.2000		-56.10	[-131.80; 19.60]	1.3%
Astinchap 2021 ET	15	158.00	56.0000	15	173.00	34.0000		-15.00	[-48.15; 18.15]	5.2%
Astinchap 2021 RT	15	175.00	7.0000	15	173.00	34.0000		2.00	[-15.57; 19.57]	10.6%
Whyte 2020	15	152.34	23.9100	12	151.45	33.6600		0.89	[-21.67; 23.45]	8.4%
Houghton 2017	12	150.57	70.8600	12	203.71	88.5700		-53.14	[-117.32; 11.04]	1.8%
Sullivan 2012	12	140.35	16.5000	6	202.40	87.4000		-62.05	[-132.60; 8.50]	1.5%
Achten 2003	4	177.14	17.7100	5	469.42	124.0000		-292.28	[-402.35; -182.21]	0.6%
Abdelbasset 2020	16	173.60	9.2000	16	200.30	11.6000		-26.70	[-33.95; -19.45]	15.9%
Hallsworth 2015	11	150.57	19.3400	12	150.57	70.8600		0.00	[-41.69; 41.69]	3.7%
Rezende 2016	19	151.56	64.1000	21	168.70	72.7000		-17.14	[-59.54; 25.26]	3.6%
Abdelbasset 2019	16	173.60	9.2000	16	200.30	11.6000		-26.70	[-33.95; -19.45]	15.9%
**Random effects model**	**341**			**284**				**-21.54**	**[-34.79; -8.29]**	**100.0%**

Heterogeneity: $I^2 = 60.1\%$, $\tau^2 = 119.4382$, $\chi^2_{19} = 47.64$ ($p = 0.0003$)

Test for overall effect: $t_{19} = -3.40$ ($p = 0.0030$)

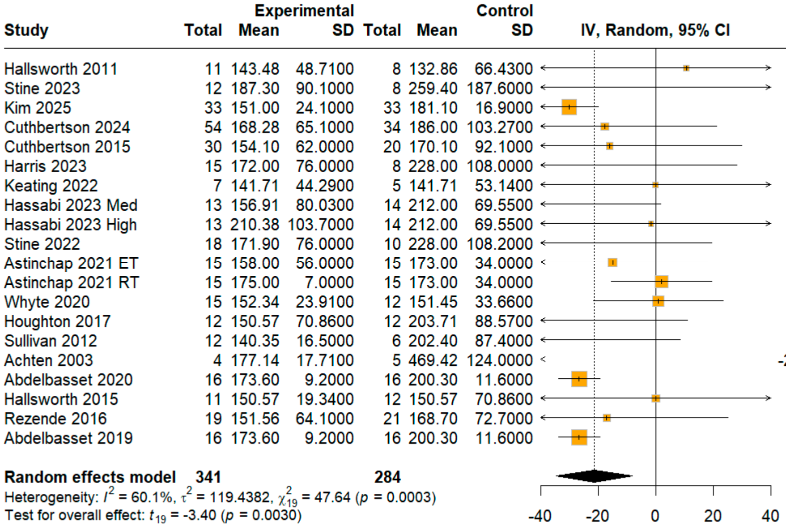

Supplementary Figure S14b. Sensitivity analysis for serum triglyceride levels, excluding non-randomized studies (Norouzpur 2021).

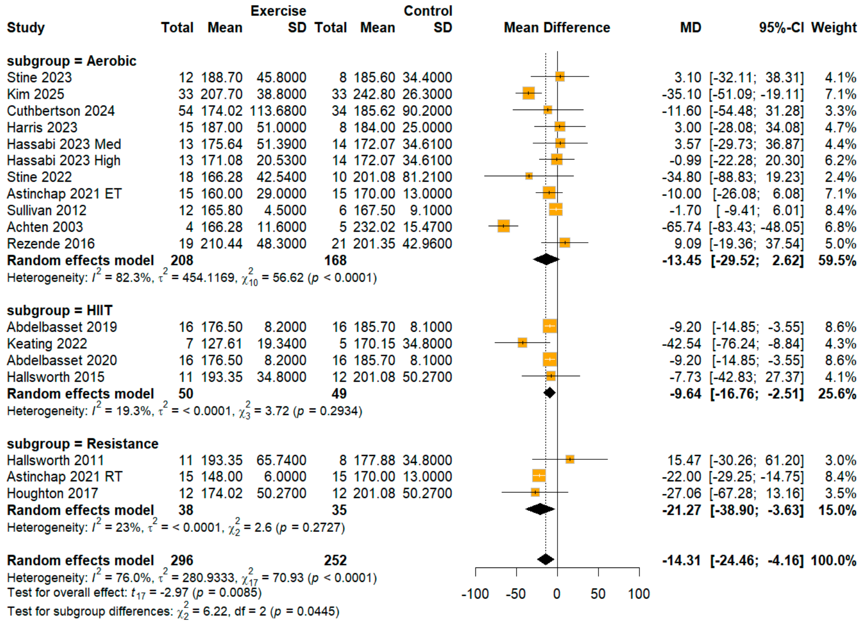

Supplementary Figure S14c. Subgroup analysis based on exercise modality for serum triglyceride levels; Aerobic=Aerobic Exercise, HIIT=High Intensity Interval Training, Resistance=Resistance Training, Mixed=A combination of any of the above three.

Study	Experimental Total	Experimental Mean	Experimental SD	Control Total	Control Mean	Control SD	MD	95%-CI	Weight
Stine 2023	12	44.50	8.5000	8	41.70	19.2000	2.80	[-11.35; 16.95]	1.9%
Kim 2025	33	52.50	8.2000	33	40.90	6.6000	11.60	[8.01; 15.19]	10.0%
Cuthbertson 2024	54	42.54	28.4386	34	38.67	22.5658	3.87	[-6.86; 14.60]	3.0%
Cuthbertson 2015	30	44.90	15.1000	20	42.50	12.4000	2.40	[-5.26; 10.06]	4.9%
Harris 2023	15	44.00	7.0000	8	40.00	12.0000	4.00	[-5.04; 13.04]	3.9%
Keating 2022	7	34.80	7.7300	5	34.80	3.8700	0.00	[-6.66; 6.66]	5.8%
Hassabi 2023 Med	13	43.82	9.6800	14	39.64	10.5300	4.18	[-3.44; 11.80]	4.9%
Hassabi 2023 High	13	42.69	8.9300	14	39.64	10.5300	3.05	[-4.30; 10.40]	5.2%
Stine 2022	18	44.00	7.1000	10	29.60	11.7000	14.40	[6.44; 22.36]	4.7%
Astinchap 2021 ET	15	45.00	9.0000	15	39.00	8.0000	6.00	[-0.09; 12.09]	6.5%
Astinchap 2021 RT	15	43.00	7.0000	15	39.00	8.0000	4.00	[-1.38; 9.38]	7.3%
Whyte 2020	15	35.96	8.1200	12	34.00	9.6700	1.96	[-4.88; 8.81]	5.7%
Sullivan 2012	12	39.90	2.5000	6	38.50	3.1000	1.40	[-1.46; 4.26]	11.2%
Abdelbasset 2020	16	39.70	3.8000	16	37.20	4.1000	2.50	[-0.24; 5.24]	11.4%
Rezende 2016	19	60.05	24.3600	21	51.05	15.1600	9.00	[-3.73; 21.73]	2.3%
Abdelbasset 2019	16	39.70	3.8000	16	37.20	4.1000	2.50	[-0.24; 5.24]	11.4%
**Random effects model**	**303**			**247**			**4.36**	**[2.29; 6.43]**	**100.0%**

Heterogeneity: $I^2 = 53.5\%$, $\tau^2 = 8.1817$, $\chi^2_{15} = 32.22$ ($p = 0.0060$)

Test for overall effect: $t_{15} = 4.49$ ($p = 0.0004$)

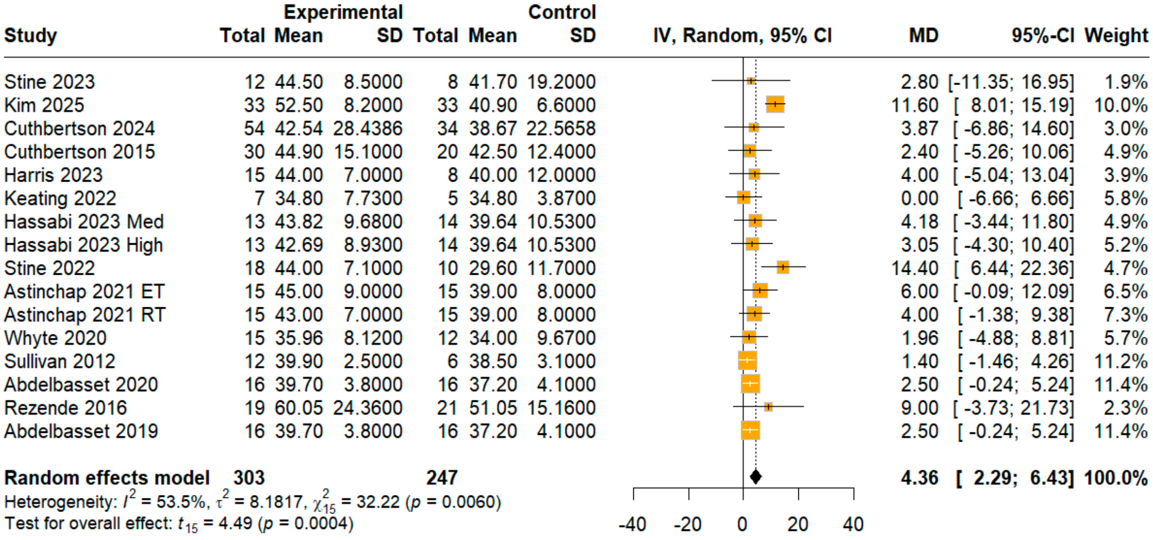

Supplementary Figure S15. Forest plot of the pooled mean difference in change in high-density lipoprotein (HDL) levels.

Study	P-value	Tau²	Tau	I²
Omitting Stine 2023	0.0007	8.4562	2.9080	56.5%
Omitting Kim 2025	0.0004	0	0	0%
Omitting Cuthbertson 2024	0.0007	8.6198	2.9360	56.6%
Omitting Cuthbertson 2015	0.0006	8.8093	2.9680	56.3%
Omitting Harris 2023	0.0008	8.7498	2.9580	56.6%
Omitting Keating 2022	0.0004	8.2983	2.8807	54.6%
Omitting Hassabi 2023 Med	0.0008	8.8985	2.9830	56.5%
Omitting Hassabi 2023 High	0.0007	8.8968	2.9827	56.5%
Omitting Stine 2022	0.0004	6.3450	2.5189	44.9%
Omitting Astinchap 2021 ET	0.0010	8.9176	2.9862	55.9%
Omitting Astinchap 2021 RT	0.0009	9.2312	3.0383	56.6%
Omitting Whyte 2020	0.0006	8.8103	2.9682	56.1%
Omitting Sullivan 2012	0.0004	8.2845	2.8783	51.1%
Omitting Abdelbasset 2020	0.0006	9.1911	3.0317	54.8%
Omitting Rezende 2016	0.0008	8.3367	2.8873	55.7%
Omitting Abdelbasset 2019	0.0006	9.1911	3.0317	54.8%
**Total (95% CI)**	**0.0004**	**8.1817**	**2.8604**	**53.5%**

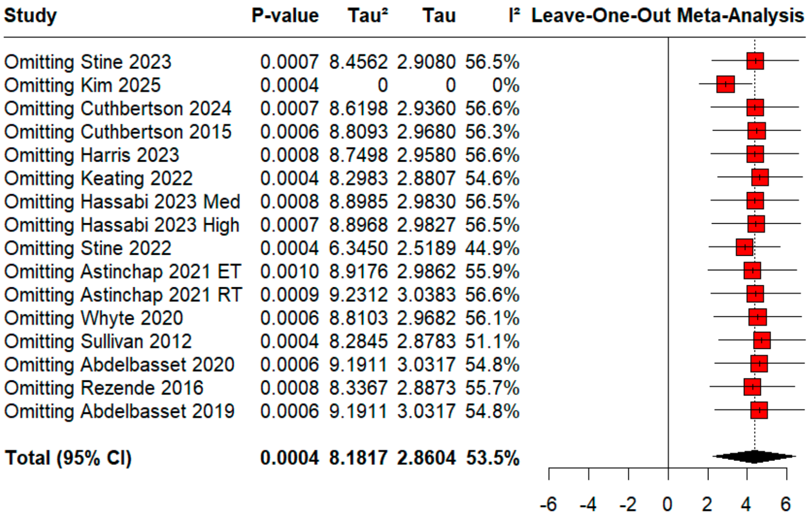

Supplementary Figure S15a. Leave-one-out sensitivity analysis for high-density lipoprotein (HDL) levels.

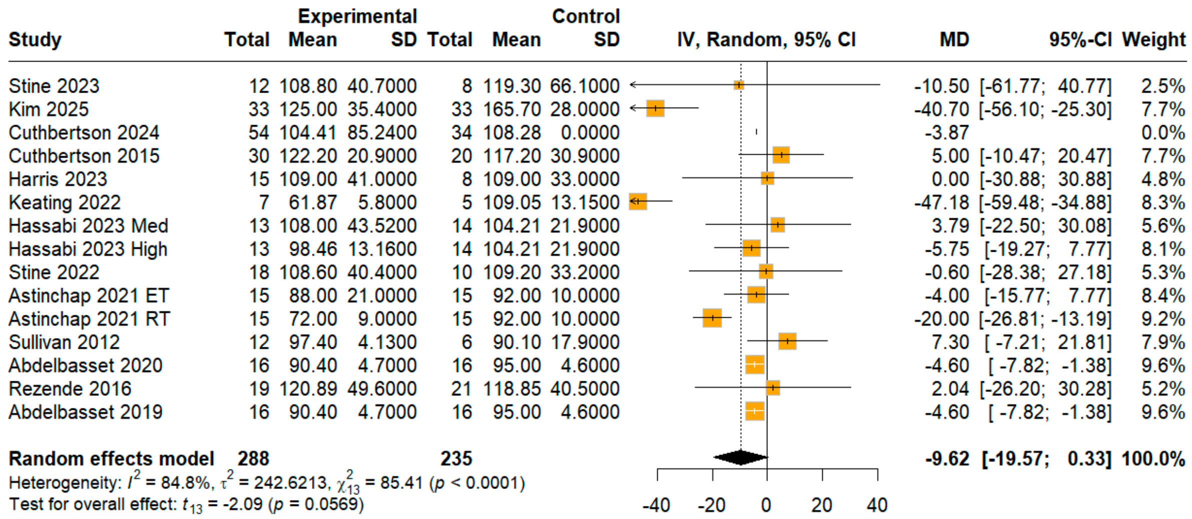

Supplementary Figure S16. Forest plot of the pooled mean difference in change in low-density lipoprotein (LDL) levels.

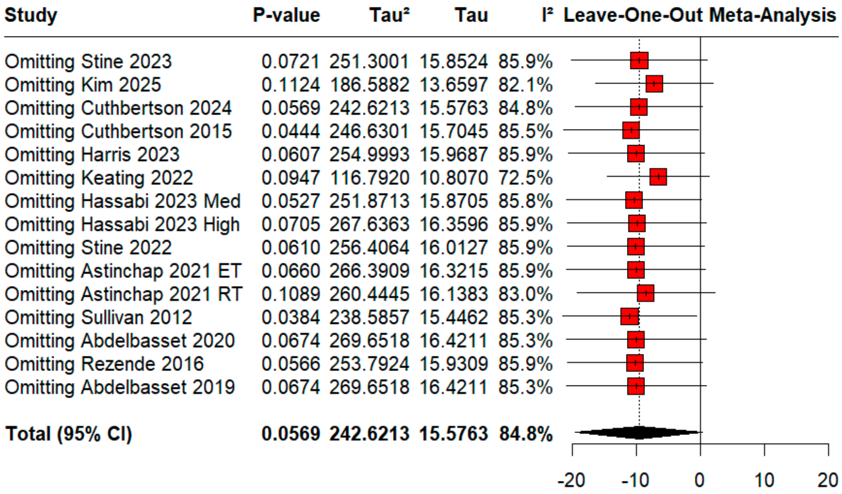

Supplementary Figure S16a. Leave-one-out sensitivity analysis for low-density lipoprotein (LDL) levels.

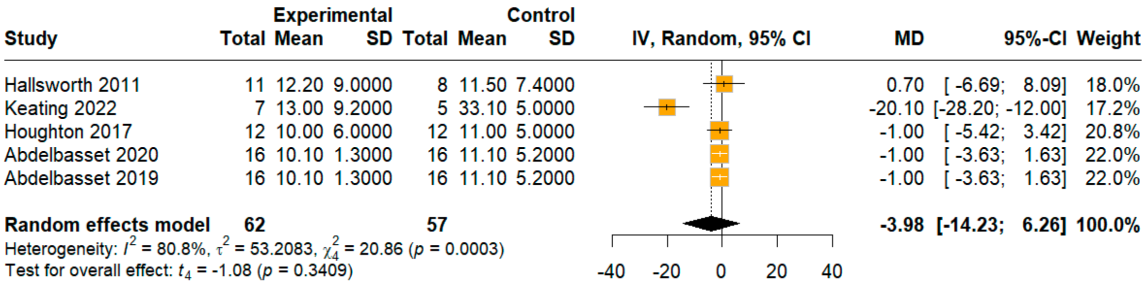

Supplementary Figure S17. Forest plot of the pooled mean difference in change in intrahepatic triglyceride levels.

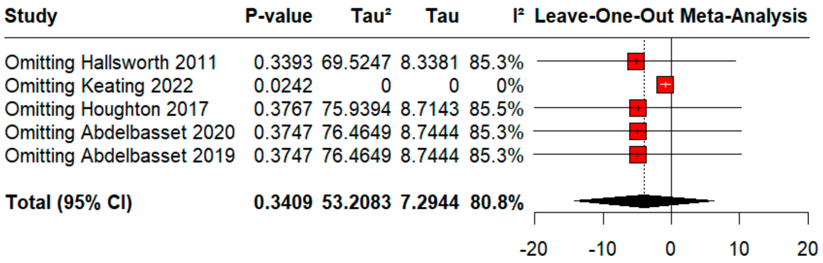

Supplementary Figure S17a. Leave-one-out sensitivity analysis for intrahepatic triglyceride levels.

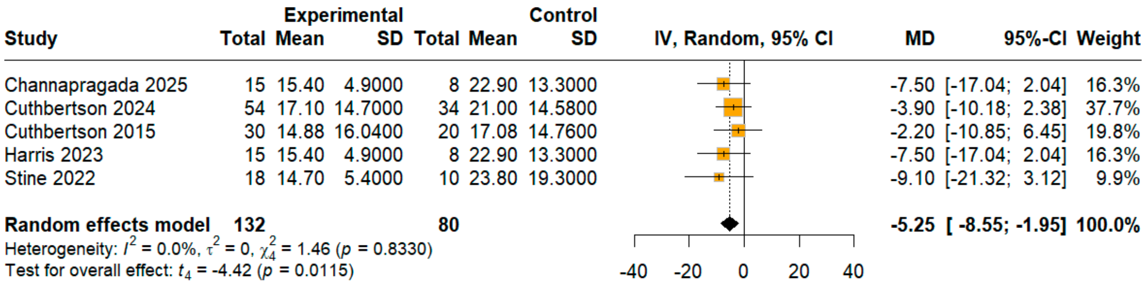

Supplementary Figure S18. Forest plot of the pooled mean difference in change in magnetic resonance imaging–proton density fat fraction (MRI-PDFF).

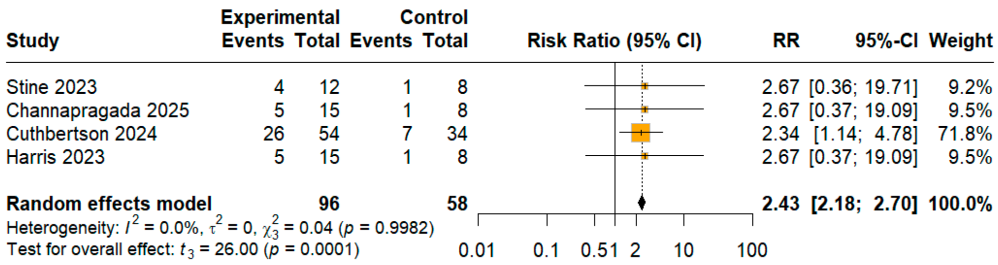

Supplementary Figure S19. Forest plot of the pooled risk ratio for achieving greater than 30% reduction in MRI-PDFF.

Section and Topic	Item #	Checklist item	Location where item is reported
**TITLE**			
Title	1	Identify the report as a systematic review.	Page 1
**ABSTRACT**			
Abstract	2	See the PRISMA 2020 for Abstracts checklist.	Page 1
**INTRODUCTION**			
Rationale	3	Describe the rationale for the review in the context of existing knowledge.	Page 2Section 1
Objectives	4	Provide an explicit statement of the objective(s) or question(s) the review	Page 2 Section

Section and Topic	Item #	Checklist item	Location where item is reported
		addresses.	1
**METHODS**			Page 3 Section 2
Eligibility criteria	5	Specify the inclusion and exclusion criteria for the review and how studies were grouped for the syntheses.	2.2
Information sources	6	Specify all databases, registers, websites, organisations, reference lists and other sources searched or consulted to identify studies. Specify the date when each source was last searched or consulted.	2.1
Search strategy	7	Present the full search strategies for all databases, registers and websites, including any filters and limits used.	2.1
Selection process	8	Specify the methods used to decide whether a study met the inclusion criteria of the review, including how many reviewers screened each record and each report retrieved, whether they worked independently, and if applicable, details of automation tools used in the process.	2.2
Data collection process	9	Specify the methods used to collect data from reports, including how many reviewers collected data from each report, whether they worked independently, any processes for obtaining or confirming data from study investigators, and if applicable, details of automation tools used in the process.	2.4
Data items	10a	List and define all outcomes for which data were sought. Specify whether all results that were compatible with each outcome domain in each study were sought (e.g. for all measures, time points, analyses), and if not, the methods used to decide which results to collect.	2.3
	10b	List and define all other variables for which data were sought (e.g. participant and intervention characteristics, funding sources). Describe any assumptions made about any missing or unclear information.	2.3
Study risk of bias assessment	11	Specify the methods used to assess risk of bias in the included studies, including details of the tool(s) used, how many reviewers assessed each study and whether they worked independently, and if applicable, details of automation tools used in the process.	2.6
Effect measures	12	Specify for each outcome the effect measure(s) (e.g. risk ratio, mean difference) used in the synthesis or presentation of results.	2.5
Synthesis methods	13a	Describe the processes used to decide which studies were eligible for each synthesis (e.g. tabulating the study intervention characteristics and comparing against the planned groups for each synthesis (item #5)).	2.4
	13b	Describe any methods required to prepare the data for presentation or synthesis, such as handling of missing summary statistics, or data conversions.	2.4
	13c	Describe any methods used to tabulate or visually display results of individual studies and syntheses.	2.5
	13d	Describe any methods used to synthesize results and provide a rationale for the choice(s). If meta-analysis was performed, describe the model(s), method(s) to identify the presence and extent of statistical heterogeneity, and software package(s) used.	2.5
	13e	Describe any methods used to explore possible causes of heterogeneity among study results (e.g. subgroup analysis, meta-regression).	2.5
	13f	Describe any sensitivity analyses conducted to assess robustness of the synthesized results.	2.5
Reporting bias	14	Describe any methods used to assess risk of bias due to missing results in a	2.6

Section and Topic	Item #	Checklist item	Location where item is reported
assessment		synthesis (arising from reporting biases).	
Certainty assessment	15	Describe any methods used to assess certainty (or confidence) in the body of evidence for an outcome.	2.5
**RESULTS**			Page 4 Section 3
Study selection	16a	Describe the results of the search and selection process, from the number of records identified in the search to the number of studies included in the review, ideally using a flow diagram.	3.1
	16b	Cite studies that might appear to meet the inclusion criteria, but which were excluded, and explain why they were excluded.	3.1
Study characteristics	17	Cite each included study and present its characteristics.	3.2
Risk of bias in studies	18	Present assessments of risk of bias for each included study.	3.3
Results of individual studies	19	For all outcomes, present, for each study: (a) summary statistics for each group (where appropriate) and (b) an effect estimate and its precision (e.g. confidence/credible interval), ideally using structured tables or plots.	3.4
Results of syntheses	20a	For each synthesis, briefly summarise the characteristics and risk of bias among contributing studies.	3.3
	20b	Present results of all statistical syntheses conducted. If meta-analysis was done, present for each the summary estimate and its precision (e.g. confidence/credible interval) and measures of statistical heterogeneity. If comparing groups, describe the direction of the effect.	3.4
	20c	Present results of all investigations of possible causes of heterogeneity among study results.	3.4
	20d	Present results of all sensitivity analyses conducted to assess the robustness of the synthesized results.	3.4
Reporting biases	21	Present assessments of risk of bias due to missing results (arising from reporting biases) for each synthesis assessed.	3.3
Certainty of evidence	22	Present assessments of certainty (or confidence) in the body of evidence for each outcome assessed.	3
**DISCUSSION**			Page 16 Section 4
Discussion	23a	Provide a general interpretation of the results in the context of other evidence.	4
	23b	Discuss any limitations of the evidence included in the review.	4
	23c	Discuss any limitations of the review processes used.	4
	23d	Discuss implications of the results for practice, policy, and future research.	4
**OTHER INFORMATION**			
Registration and protocol	24a	Provide registration information for the review, including register name and registration number, or state that the review was not registered.	2.1
	24b	Indicate where the review protocol can be accessed, or state that a protocol was not prepared.	2.1
	24c	Describe and explain any amendments to information provided at registration or in the protocol.	2.1
Support	25	Describe sources of financial or non-financial support for the review, and the role of the funders or sponsors in the review.	Page 20

Section and Topic	Item #	Checklist item	Location where item is reported
Competing interests	26	Declare any competing interests of review authors.	Page 20
Availability of data, code and other materials	27	Report which of the following are publicly available and where they can be found: template data collection forms; data extracted from included studies; data used for all analyses; analytic code; any other materials used in the review.	Page 20

Table S1: PRISMA Checklist for our study.
